# Differentially disrupted spinal cord and muscle energy metabolism in spinal and bulbar muscular atrophy

**DOI:** 10.1172/jci.insight.178048

**Published:** 2024-03-07

**Authors:** Danielle DeBartolo, Frederick J. Arnold, Yuhong Liu, Elana Molotsky, Hsin-Yao Tang, Diane E. Merry

**Affiliations:** 1 Department of Biochemistry and Molecular Biology, Sidney Kimmel Medical College, Thomas Jefferson University, Philadelphia, Pennsylvania, USA.; 2 Proteomics and Metabolomics Shared Resource, Molecular and Cellular Oncogenesis Program, The Wistar Institute, Philadelphia, Pennsylvania, USA.

**Keywords:** Genetics, Neuroscience, Mouse models, Neuromuscular disease, Proteomics

## Abstract

Prior studies showed that polyglutamine-expanded androgen receptor (AR) is aberrantly acetylated and that deacetylation of the mutant AR by overexpression of nicotinamide adenine dinucleotide–dependent (NAD^+^-dependent) sirtuin 1 is protective in cell models of spinal and bulbar muscular atrophy (SBMA). Based on these observations and reduced NAD^+^ in muscles of SBMA mouse models, we tested the therapeutic potential of NAD^+^ restoration in vivo by treating postsymptomatic transgenic SBMA mice with the NAD^+^ precursor nicotinamide riboside (NR). NR supplementation failed to alter disease progression and had no effect on increasing NAD^+^ or ATP content in muscle, despite producing a modest increase of NAD^+^ in the spinal cords of SBMA mice. Metabolomic and proteomic profiles of SBMA quadriceps muscles indicated alterations in several important energy-related pathways that use NAD^+^, in addition to the NAD^+^ salvage pathway, which is critical for NAD^+^ regeneration for use in cellular energy production. We also observed decreased mRNA levels of nicotinamide riboside kinase 2 (*Nmrk2*), which encodes a key kinase responsible for NR phosphorylation, allowing its use by the NAD^+^ salvage pathway. Together, these data suggest a model in which NAD^+^ levels are significantly decreased in muscles of an SBMA mouse model and intransigent to NR supplementation because of decreased levels of *Nmrk2*.

## Abstract 2

Spinal and bulbar muscular atrophy model mice exhibit NAD^+ ^deficiency; however, nicotinamide riboside treatment does alleviate symptoms in muscle due to decreased Nmrk2.

## Introduction

Spinal and bulbar muscular atrophy (SBMA) is an X-linked, neuromuscular disease caused by a polyglutamine (polyQ) repeat expansion in the androgen receptor (AR) (1). In accordance with disease pathology, which is characterized by a loss of lower motor neurons in the brain stem and spinal cord (2, 3) as well as by cell-autonomous toxicity in muscle (4, 5), patients with SBMA predominantly present with neuromuscular symptoms, such as weakness and cramping of muscles, tremor, dysphagia, and dysarthria (2, 6–10). However, metabolic disturbances have also been reported in SBMA, with a number of recent studies greatly expanding our understanding of and appreciation for the system-wide effects of mutant AR. Notably, impaired glucose homeostasis is now understood to be a common feature of SBMA (11–13). Although there is conflicting evidence for whether fasting blood glucose levels are normal (14), low (13), or high (11, 12, 15) in patients with SBMA, there is agreement regarding an increase in insulin resistance, as measured by the Homeostatic Model Assessment of Insulin Resistance (12, 13, 15). Moreover, there is evidence that insulin resistance correlates with motor dysfunction in patients with SBMA patients (13), highlighting the therapeutic possibility of improving motor function by treating metabolic disturbances downstream of the mutant AR.

The AR is a member of the steroid hormone receptor subfamily of the nuclear receptor superfamily. In the absence of ligand, the AR resides in the cytoplasm in an inactive aporeceptor complex that contains chaperones (HSC70, HSP40, HSP90, HIP, HOP), P23, and immunophilins (CYP40, FKBP51, FKBP52) (16, 17). Upon binding of testosterone or dihydrotestosterone, the AR undergoes a conformational change inducing nuclear translocation and the transcriptional regulation of target genes. Ultimately, the AR is degraded by the ubiquitin-proteasome system in the cytoplasm following nuclear export (18–23). Throughout its cellular life cycle, both AR trafficking and function are regulated by posttranslational modifications, several of which are capable of modulating the toxicity of polyQ-expanded AR (24–27). Indeed, we have reported that deacetylation of the mutant AR at lysines 631, 633, and 634 by overexpression of the deacetylase sirtuin 1 (SIRT1) has a substantial protective effect in cell models of SBMA (25).

In addition to deacetylating the AR, SIRT1 plays a key role in several pathways known to be disrupted in SBMA, such as glucose metabolism (28) and mitochondrial function (29). Given that SIRT1 activity affects cellular pathways associated with SBMA via both AR-specific and nonspecific mechanisms, increasing the activity of SIRT1 could be therapeutically beneficial at multiple levels of disease pathophysiology. As an NAD^+^-dependent enzyme, SIRT1 activity can be effectively increased by dietary supplementation with NAD^+^ precursors, most commonly niacin (NA) or nicotinamide (NAM) (30). There is evidence that the *K_m_* of SIRT1 for NAD^+^ is around the physiological concentration of free NAD^+^ in the nucleus (31, 32), suggesting that NAD^+^ levels can rate-limit SIRT1 activity (32, 33). Thus, a decrease in NAD^+^ levels, as occurs during normal aging (34–39), as well as in a mouse model of SBMA after exercise (40), can correspondingly attenuate SIRT1 activity. The fact that overexpression of SIRT1 is protective in mouse models of Alzheimer’s disease (41), amyotrophic lateral sclerosis (ALS) (41, 42), and Huntington’s disease (43, 44) further indicates a central role for SIRT1 in age-associated neurodegenerative disorders.

Intracellularly, NAD(H) cycles between its oxidized (NAD^+^) and reduced (NADH) forms. This process is regulated by cellular energy production, as NAD^+^ is reduced during glycolysis, while NADH is oxidized during oxidative phosphorylation. The ratio of NAD^+^/NADH varies between tissues and cell types, but notably, one recent study determined NAD^+^/NADH to be approximately 4.8 in the human brain, with increasing age correlating with a lower NAD^+^/NADH ratio (38). In addition to changes in its redox state, NAD^+^ concentration is determined by its relative rates of synthesis and consumption. NAD^+^ is synthesized de novo from tryptophan via the kynurenine pathway (45) and recycled by several salvage pathways, which generate NAD^+^ from NA via the Preiss-Handler pathway (46, 47), from nicotinamide riboside (NR) via the nicotinamide ribose kinase pathway (48), or from NAM via the activity of nicotinamide phosphoribosyltransferase (NAMPT). Several distinct families of enzymes consume NAD^+^, notably the SIRT family of deacetylases, poly (ADP-ribose) polymerases (PARPs), and cyclic ADP-ribose hydrolases (CD38 and CD157). Use of NAD^+^ by these enzymes generates NAM, which can be recycled into NAD^+^ as described.

Our study sought to evaluate the therapeutic potential of increasing SIRT1 activity in a transgenic mouse model of SBMA via supplementation with the NAD^+^ precursor NR (48). NR has been shown to boost SIRT1 activity in a number of cell and animal models (39, 49–52) and, importantly, increases NAD^+^ content in tissues relevant to SBMA, notably in skeletal muscle (39, 50, 53) and in the brain (54, 55). By evaluating the effect of NR supplementation on behavior and pathology in an SBMA mouse model, this study provides new insights into the therapeutic potential of activating SIRT1 via NR in SBMA and expands our understanding of energy metabolism in tissues relevant to this disease. Moreover, our combined analyses of metabolomic and proteomic alterations in muscle of an SBMA mouse model yield new insights into dysfunctional metabolic pathways in SBMA and suggest new avenues for therapeutic development.

## Results

### Dietary supplementation with NR does not ameliorate motor dysfunction or muscle pathology in a mouse model of SBMA.

**Figure 1:**
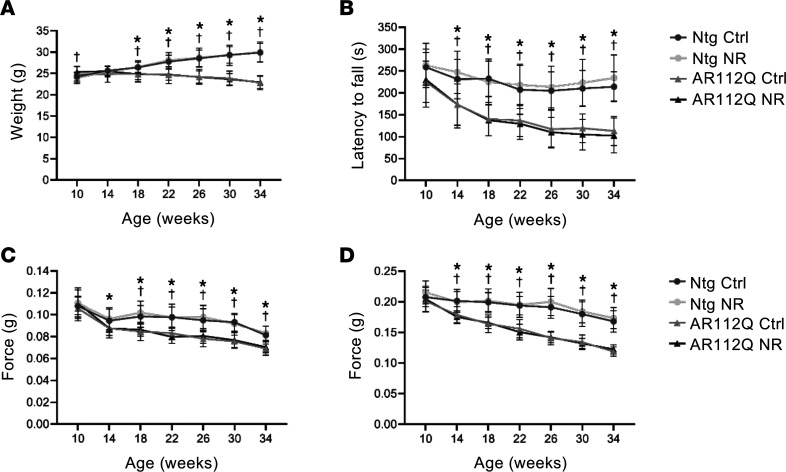
Dietary supplementation with NR does not ameliorate motor dysfunction in a mouse model of SBMA. (**A**) Body weight analysis of nontransgenic mice fed control chow (*n* = 24), nontransgenic mice fed chow supplemented with 400 mg/kg/d NR (*n* = 26), AR112Q mice fed control chow (*n* = 26), and AR112Q mice fed chow supplemented with 400 mg/kg/d NR (*n* = 24). (**B**) Accelerating rotarod analysis of control or NR-treated nontransgenic and AR112Q mice. Mice performed 4 trials every 4 weeks beginning at 10 weeks of age. Forepaw (**C**) and all-paw (**D**) grip strength of control or NR-treated nontransgenic and AR112Q mice. Mice were tested every 4 weeks beginning at 10 weeks of age. Statistical significance for all behavioral testing was determined using mixed effects analysis with post hoc Tukey test for multiple comparisons. Data represent mean ± SD. *P < 0.05 AR112Q Ctl versus Ntg Ctl and Ntg NR; ^†^*P* < 0.05 AR112Q NR versus Ntg Ctl and Ntg NR.

To determine the effect of NR treatment on disease progression in a mouse model of SBMA, we used transgenic mice expressing AR112Q under the control of the prion protein (PrP) promoter (56). The PrP-AR112Q mouse model of SBMA has been well characterized and recapitulates key aspects of the human disease, such as slowly progressive motor dysfunction and mutant AR aggregation (57–59). In addition to the substantial overexpression of the AR transgene in the nervous system, this mouse model also expresses the mutant AR transgene in muscle, at a level closer to that of endogenous AR. A number of studies have shown that dietary supplementation of 400 mg/kg/d NR is well tolerated in mice and is capable of changing metabolic and/or phenotypic outcomes (39, 50, 52, 53, 60). To assess NR as a therapy for SBMA, age-matched cohorts of male nontransgenic and AR112Q mice were treated with 400 mg/kg/d NR (in chow) after the onset of symptoms in transgenic AR112Q mice (as determined by latency to fall from an accelerating rotarod). Previous studies have shown that PrP-AR112Q mice develop rotarod deficits between 8 weeks (58) and 12 weeks (57, 59) of age. In the present study, transgenic mice exhibited rotarod deficits at 10 weeks of age (Figure 1B), and NR treatment commenced immediately following behavioral testing at 10 weeks. As shown in Figure 1, treatment with NR had no effect on the weight or motor function of nontransgenic or AR112Q mice. Both control and NR-treated AR112Q mice exhibited slowly progressive weight loss (Figure 1A), rotarod deficits (Figure 1B), and muscle weakness (Figure 1, C and D) relative to both treated and untreated nontransgenic mice. This result may indicate that NAD^+^ synthesis or energy metabolism downstream of NR is compromised in postsymptomatic SBMA mice.

**Figure 2:**
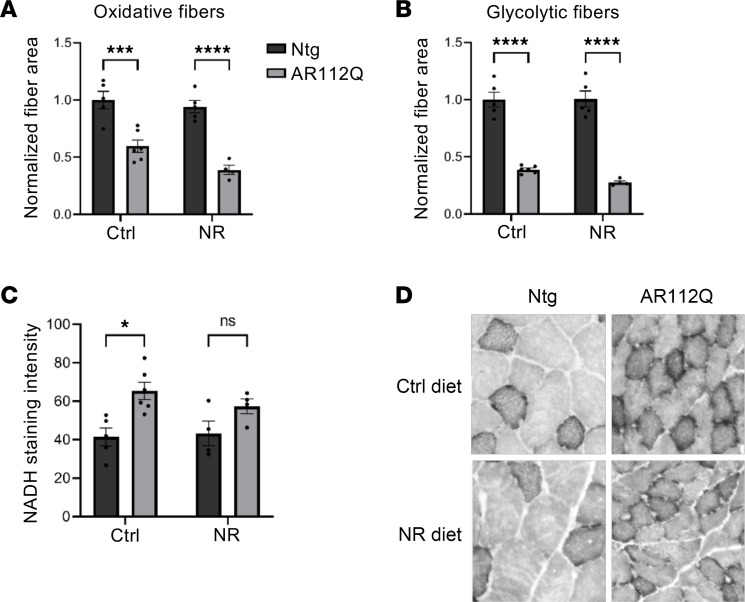
Dietary supplementation with NR does not ameliorate muscle pathology in a mouse model of SBMA. (**A** and **B**) Cross-sectional area of oxidative (**A**) and glycolytic (**B**) muscle fibers from the quadriceps of nontransgenic mice fed control chow (*n* = 5), nontransgenic mice fed chow supplemented with 400 mg/kg/d NR (*n* = 5), AR112Q mice fed control chow (*n* = 6), and AR112Q mice fed chow supplemented with 400 mg/kg/d NR (*n* = 4) at 36 weeks. Statistical significance was determined by 2-way ANOVA with post hoc Tukey test. ****P < 0.0001, ***P < 0.001. (**C**) NADH-diaphorase staining intensity of muscle fibers from quadriceps muscles of nontransgenic mice fed control chow (*n* = 5), nontransgenic mice fed chow supplemented with NR (*n* = 4), AR112Q mice fed control chow (*n* = 6), and AR112Q mice fed chow supplemented with NR (*n* = 4) at 36 weeks. Statistical significance was determined by 2-way ANOVA with post hoc Tukey test. Data represent mean ± SEM. *P < 0.05. (**D**) Representative images of NADH-diaphorase–stained quadriceps muscle from control or NR-treated nontransgenic and AR112Q mice at 36 weeks. All images taken at original magnification, 5×.

At 36 weeks of age, quadriceps (quad) muscle was collected from each cohort and analyzed for fiber size as well as for fiber type switching. A glycolytic to oxidative shift has been reported in the muscles of multiple mouse models of SBMA (4, 61–64), as well as in patients with SBMA (65). In mice, this correlates with a shift toward oxidative metabolism and disrupted glycolysis in muscle (40, 61). Here, we found that both oxidative and glycolytic muscle fibers (identified by NADH-diaphorase staining) were smaller in AR112Q male mice than in nontransgenic male mice (Figure 2, A and B). Additionally, in agreement with other SBMA mouse models, glycolytic fibers appeared to be more vulnerable than oxidative fibers in PrP-AR112Q mice, as reflected by a more substantial decrease in the size of glycolytic fibers compared with oxidative fibers (Figure 2, A and B). NR treatment had no effect on the muscle fiber size of either nontransgenic or AR112Q mice (Figure 2, A and B). We also observed an oxidative shift in the muscle fibers of control AR112Q mice compared with control nontransgenic mice, as determined by NADH staining intensity (Figure 2, C and D). Although there was no significant difference between the intensity of NADH staining in the quad of NR-treated AR112Q mice compared with NR-treated nontransgenic mice, NADH staining in these mice was not statistically reduced compared with control AR112Q mice. Thus, treatment of postsymptomatic AR112Q mice with 400 mg/kg/d NR had no effect on the progression of motor symptoms or on muscle pathology in the present study. Importantly, however, we found, as recently shown (64), that the PrP-AR112Q mouse model, which expresses high levels of the AR transgene in the CNS and close to endogenous levels in muscle, exhibits the same glycolytic to oxidative shift found in the muscles of other mouse models of SBMA.

### NR treatment does not affect AR aggregation in SBMA mice.

Overexpression of SIRT1 was previously shown to reduce the aggregation of polyQ-expanded AR in a PC12 cell model of SBMA (25). As NR is known to increase the activity of SIRT1 in vivo (39, 50–52), we hypothesized that NR-treated AR112Q mice would form fewer intranuclear inclusions of aggregated AR than untreated mice. Immunofluorescence analysis of spinal cord motor neurons revealed no effect of NR treatment on AR aggregation (Supplemental Figure 1, A and B; supplemental material available online with this article; https://doi.org/10.1172/jci.insight.178048DS1).

### NAD^+^ and ATP levels are substantially reduced in the muscle but not in the spinal cord of SBMA mice.

NR has been shown to increase NAD^+^ levels in both the CNS (54, 55) and in the skeletal muscle (39, 50, 53) of mice. Correspondingly, NR treatment has also been shown to increase ATP content in the muscles of mice (39). Intracellular production of ATP is directly linked to NAD^+^ availability, as NAD^+^ is required for the conversion of glyceraldehyde 3-phosphate to 1, 3-bisphosphoglycerate by GAPDH during glycolysis and for the activity of 3 NAD^+^-dependent dehydrogenases in the tricarboxylic acid (TCA) cycle.

**Figure 3:**
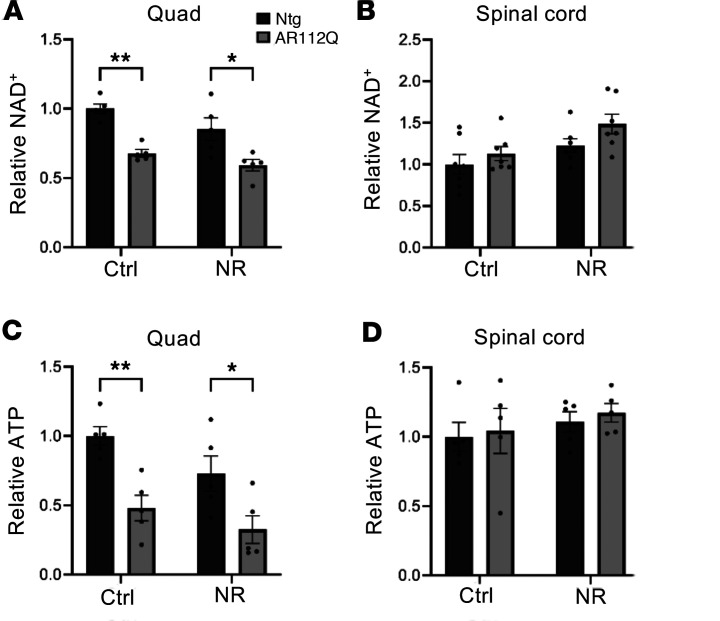
NAD^+^ and ATP levels are substantially reduced in the muscle but not in the spinal cord of SBMA mice. (**A** and **B**) Relative NAD^+^ concentrations from quadriceps muscle (**A**) (*n* = 5 per group) or spinal cord (**B**) (*n* = 7 per group) of control or NR-treated nontransgenic and AR112Q mice at 36 weeks. Statistical significance was determined by 2-way ANOVA with post hoc Tukey test. **P < 0.01, *P < 0.05. (**C** and **D**) Relative ATP concentrations from the quadriceps muscle (**C**) (*n* = 5 per group) or spinal cord (**D**) (*n* = 5 per group) of control or NR-treated nontransgenic and AR112Q mice at 36 weeks. Statistical significance was determined by 2-way ANOVA with post hoc Tukey test. Data represent mean ± SEM. **P < 0.01, *P < 0.05.

To determine the effect of NR treatment on NAD^+^ and ATP levels in the CNS and muscle, we analyzed the concentration of both metabolites in the quad and spinal cord of nontransgenic and AR112Q mice at 36 weeks of age. As shown in Figure 3, transgenic mice fed a control diet had substantially reduced NAD^+^ and ATP levels in the quad relative to nontransgenic mice fed a control diet, and supplementation with NR did not restore the content of either metabolite in the quad (Figure 3, A and C). Surprisingly, a different effect was observed in the spinal cord, as there was no difference in the NAD^+^ or ATP levels of AR112Q mice fed a control diet compared with nontransgenic mice fed a control diet (Figure 3, B and D). Following NR treatment, NAD^+^ levels in the spinal cord trended toward an increase in transgenic AR112Q mice (*P* = 0.086) while NR had no effect on ATP levels (Figure 3, B and D).

These results suggest that energy metabolism is disrupted in the muscle, but not in the spinal cord, of SBMA mice. Moreover, the intransigence of this disruption to NR supplementation suggested to us that NAD^+^ and/or ATP synthesis pathways are disrupted downstream of NR or that NR entry into the NAD^+^ salvage pathway is altered in SBMA.

### PrP-AR112Q mice exhibit polydipsia and hypoglycemia.

A number of studies have demonstrated that impaired glucose homeostasis is a common feature of SBMA (11–13). Moreover, a recent study found a significant correlation between insulin resistance and motor dysfunction in a cohort of patients with SBMA (13), highlighting the clinical relevance of glucose homeostasis in SBMA. Interestingly, PrP-AR112Q mice exhibit polydipsia (Supplemental Figure 2A), a common symptom of untreated diabetes mellitus, and thus, may indicate hyperglycemia. However, we found that the blood glucose of AR112Q mice was markedly lower than nontransgenic mice (Supplemental Figure 2B). While this result was unexpected, it is possible that these metabolic disturbances are unrelated, as polydipsia may also be a symptom of diabetes insipidus and, therefore, unrelated to blood glucose levels. Whether PrP-AR112Q mice exhibit pathology in the hypothalamus, pituitary gland, and/or kidneys to cause this condition remains to be further investigated.

Hypoglycemia in AR112Q mice may be caused by a decrease in SIRT1 activity, as SIRT1 is known to promote gluconeogenesis through the deacetylation of PGC1A (28), STAT3 (66), and FOXO1 (67). The role of SIRT1 in regulating glucose homeostasis is complex, however, as SIRT1 also promotes glucose-stimulated insulin secretion (68, 69), and NAD^+^ precursors have been shown to increase insulin sensitivity in mice via SIRT1 activation (36, 70). Given the importance of SIRT1 activity in regulating glucose homeostasis at a number of levels, we hypothesized that NR treatment could normalize blood glucose levels in AR112Q mice via the activation of SIRT1. As shown in Supplemental Figure 2B, dietary supplementation with NR had no effect on the blood glucose levels of SBMA mice. This correlates with an inability of NR to increase NAD^+^ content in the muscle of these mice (Figure 3A), which may be indicative of a lack of effect in other peripheral tissues, such as the liver. Taken together, we found that PrP-AR112Q mice exhibit system-wide metabolic disturbances, including impaired glucose homeostasis, which mirrors a phenotype observed in patients with SBMA. While the mechanistic basis for these phenotypes requires further investigation, it is possible that reduced SIRT1 activity caused by low NAD^+^ levels in peripheral tissues impairs gluconeogenesis in SBMA mice.

### Quad from transgenic AR112Q mice displays an altered metabolite profile that is not significantly affected by NR treatment.

**Figure 4:**
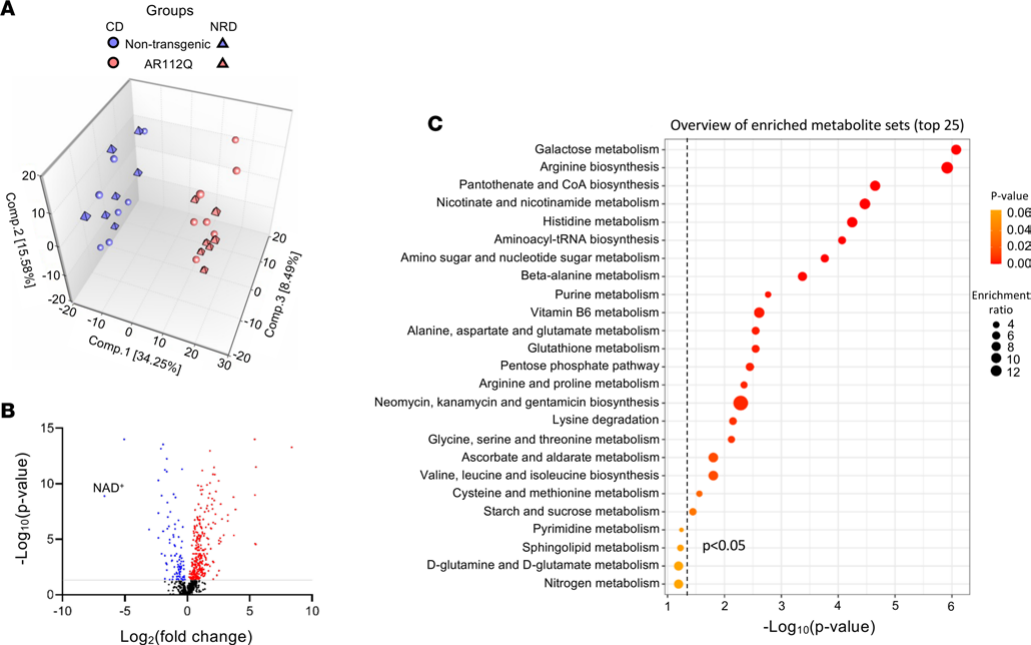
NR treatment does not significantly alter the metabolome of AR112Q mice. (**A**) Principal component analysis (PCA) of metabolomics data sets of quadriceps muscle from AR112Q (pink symbols) and nontransgenic (blue symbols) mice, treated with NR diet (NRD) or control diet (CD) (*n* = 7 per condition/genotype). Plots of nontransgenic and AR112Q mice are clearly distinguished on the first principal component axis (*x* axis). (**B**) Volcano plot depicting significantly changed metabolites (red significantly increased, blue significantly decreased) in the AR112Q quadriceps muscle, compared with nontransgenic quadriceps muscle, treated with CD. Based on *P* ≤ 0.05 (1-way ANOVA with FDR correction), false discovery rate of *q* < 0.1, and metabolite ratio of ≥1.0 for up and <1.0 for down. (**C**) Metabolite set enrichment analysis identifying overrepresented metabolic pathways based on significantly changed metabolites in AR112Q quadriceps muscle from mice on CD. Top enriched metabolite sets include galactose metabolism, amino acid metabolism, pantothenate and CoA biosynthesis, and nicotinate and nicotinamide metabolism.

To gain insight into why supplementation with NR had no effect on motor dysfunction or muscle NAD^+^ levels in transgenic AR112Q mice, we analyzed the global metabolite profile using untargeted metabolomics of spinal cord and quad from nontransgenic and transgenic AR112Q mice fed either control diet or a diet supplemented with NR. Principal component analysis (PCA) analysis of spinal cord tissue did not show a clear separation between AR112Q and nontransgenic mice regardless of treatment (Supplemental Figure 3A). In contrast, PCA showed a distinct separation of the metabolite profiles of quads from nontransgenic and AR112Q mice regardless of being fed the control diet or diet supplemented with NR (Figure 4A). Due to this distinct tissue difference, we focused on metabolism changes within the quad for the remainder of the study.

The lack of effect of NR on motor function and pathology was consistent with a lack of NR effect observed in the PCA of metabolite profiles of quads (Figure 4A). We also did not observe increased levels of NAD^+^ or NAM in nontransgenic quad following NR supplementation (Supplemental Figure 4). However, we did observe significant increases in all 4 metabolites associated with NAM clearance (nicotinamide N-oxide, 1-methylnicotinamide, N1-Methyl-2-pyridone-5-carboxamide, and N1-Methyl-4-pyridone-3-carboxamide). As NAD^+^ levels are tightly controlled, this suggests excess NAD^+^ being cleared to maintain NAD^+^ homeostasis. Given that we did not observe a significant impact of NR supplementation on the global metabolome of transgenic AR112Q quadriceps, we limited further analyses to changes between nontransgenic and transgenic AR112Q mice fed the control diet. In total, we detected 691 metabolites of which 386 were significantly changed in AR112Q quad, with 293 metabolites increased and 93 metabolites decreased. Approximately 56% of detected metabolites in the AR112Q quad showed significant differences compared with those of nontransgenic mice. Only metabolites exhibiting *P* ≤ 0.05 and *q* < 0.1 as an estimate of false discovery rate were considered for analysis. A full list of changed metabolites is available in Supplemental Table 1. Metabolites with significantly increased and decreased abundance in the transgenic AR112Q quadriceps muscle compared with nontransgenic muscle are depicted in the volcano plot (Figure 4B), with NAD^+^ being one of the metabolites with the largest decreases in AR112Q muscle.

We next grouped the significantly changed metabolites into higher order classification categories, with lipid (148 metabolites), amino acid (104 metabolites), and carbohydrate (39 metabolites) being the categories with the most altered metabolites (Supplemental Figure 3B). Interestingly, metabolites with the highest fold-changes (both increased and decreased) are involved in NAD metabolism, glycolysis, and amino acid metabolism (Supplemental Table 1).

To identify metabolic pathways associated with the altered metabolites in the transgenic AR112Q quad, we utilized metabolite set enrichment analysis (MSEA), which identifies overrepresented metabolic pathways among the changed metabolites. As several metabolic pathways contained both increased and decreased metabolites, we combined increased and decreased metabolites for analysis. The top enriched pathways included galactose metabolism, arginine biosynthesis, pantothenate and CoA biosynthesis, and nicotinate and nicotinamide metabolism (Figure 4C). In addition, amino acid metabolism of several amino acids, aminoacyl-tRNA biosynthesis, pentose phosphate pathway, vitamin B_6_, and amino sugar and nucleotide sugar metabolism pathways were also significantly overrepresented in the data set (Figure 4C).

Together, these data suggest significant global metabolic alterations in quad of transgenic AR112Q compared with nontransgenic mice, which were not significantly affected by NR supplementation. In addition, NAD^+^ was one of the top most significantly decreased metabolites, and nicotinate and nicotinamide metabolism was one of the top overrepresented metabolic pathways in AR112Q muscle, suggesting likely dysfunction in NAD^+^ metabolism, which could significantly impact energy metabolism.

### Quad from transgenic AR112Q mice displays an altered proteome profile.

**Figure 5:**
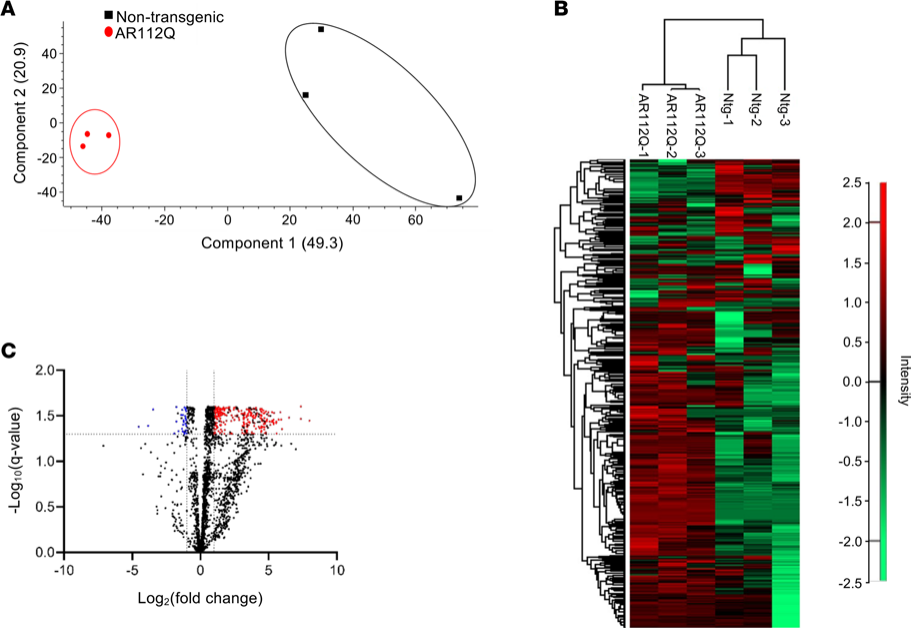
Proteome analysis of quadriceps muscle from transgenic AR112Q and nontransgenic mice reveals distinct proteomic signatures. (**A**) PCA of LC-MS/MS proteomic analysis of quadriceps muscle from AR112Q and nontransgenic mice (*n* = 3 per genotype). Plots of nontransgenic (black symbols) and AR112Q mice (red symbols) are clearly distinguished on the first principal component axis (*x* axis). (**B**) Hierarchical clustering of identified proteins with high protein intensities shown in red and low protein intensities shown in green. (**C**) Volcano plot illustrating the differentially abundant proteins (red significantly increased, blue significantly decreased) in the AR112Q quadriceps muscle compared with nontransgenic quadriceps muscle. Based on adjusted *P* ≤ 0.05 determined using the permutation method, a fold-change of 2, identified by a minimum of 2 razor+ unique peptides in at least 1 of the samples, and detected in at least 2 of the replicates. “Razor+ unique peptides” are defined as shared (nonunique) peptides assigned to the protein group with the most other peptides (Occam’s razor principle).

After identifying changed metabolites and their associated metabolic pathways, we next asked if proteins related to these pathways were altered in the quad from transgenic AR112Q mice. To evaluate changes in protein abundance, quad protein extracts from 9-month-old transgenic AR112Q and nontransgenic mice fed a control diet were analyzed by liquid chromatography-tandem mass spectrometry (LC-MS/MS) combined with label-free quantification (LFQ). In total, 26,524 peptides were identified, which could be assigned to 2,582 protein groups at a false discovery rate < 0.01. PCA (Figure 5A) of detected proteins revealed a clear separation between the protein expression profiles of quad from transgenic AR112Q and nontransgenic mice. Unsupervised hierarchical clustering of LFQ values for each sample revealed distinct clusters (Figure 5B) between AR112Q and nontransgenic quad. Based on our criteria of an adjusted *P* < 0.05 and minimum fold-change of |2|, we identified 302 significantly changed proteins with 273 upregulated and 29 downregulated in the transgenic AR112Q quad. Proteins with significantly increased and decreased abundance are depicted in the volcano plot (Figure 5C). All significantly changed proteins are listed in Supplemental Table 2.

**Figure 6:**
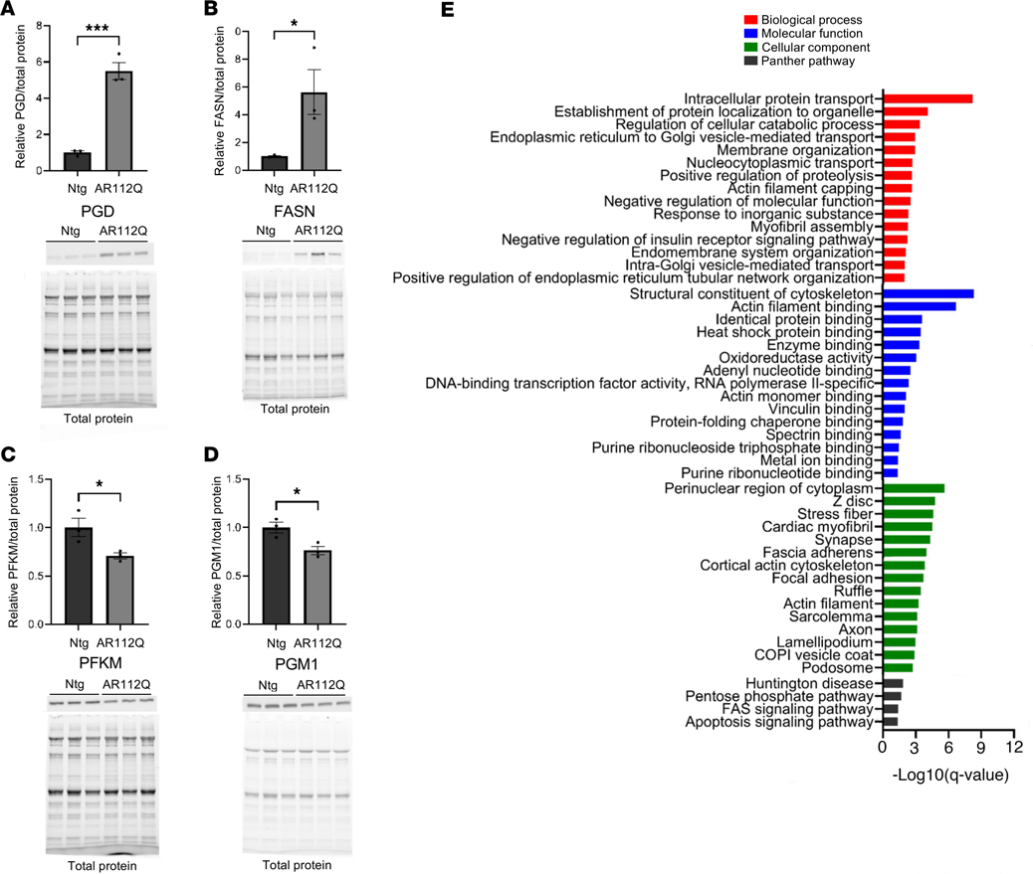
Immunoblot analysis of selected proteins and gene ontology enrichment analysis of differentially expressed proteins. (**A**–**D**) Quantification and associated Western blots for selected proteins based on either the permutation or Benjamini-Hochberg false discovery rate in AR112Q quadriceps muscle. Phosphogluconate dehydrogenase (PGD), fatty acid synthase (FASN), phosphofructokinase (PFKM), and phosphoglucomutase 1 (PGM1). Statistical significance was determined by Student’s *t* test. Data represent mean ± SEM. *n* = 3 per genotype. ***P ≤ 0.001, *P ≤ 0.05. (**E**) Gene ontology (GO) enrichment analysis of differentially expressed proteins in the transgenic AR112Q quadriceps muscle. GO biological process (red), molecular function (blue), cellular component (green), and Protein Analysis through Evolutionary Relationships (PANTHER) pathway (black) enrichment analyses of upregulated proteins performed using PANTHER bioinformatics software. Top 15 significantly enriched GO terms are shown with false discovery rate < 0.05.

To validate our proteomics data, we selected 4 significantly changed proteins (2 increased and 2 decreased) for Western blotting. Western blotting of phosphogluconate dehydrogenase (PGD), fatty acid synthase (FASN), phosphofructokinase (PFKM), and phosphoglucomutase 1 (PGM1) showed similar patterns in the transgenic AR112Q quad to that observed in the mass spectrometry data set (Figure 6, A–D). To our knowledge, this is the first global proteomic data set of quad from SBMA mice. The knockin AR113Q mouse model of SBMA displays many of the same characteristics as the transgenic PrP-AR112Q mouse model, including motor dysfunction and metabolic abnormalities. To determine if quad from the knockin AR113Q model displayed the same proteomic changes as in the transgenic model, we performed Western blotting of the same proteins and found them to be similarly changed in the quad from the knockin AR113Q mice (Supplemental Figure 5, A–C). This result suggests that knockin mice share some similar proteomic changes with transgenic AR112Q mice. The observed imbalance of increased and decreased proteins is interesting, as more proteins are increased than are decreased in the transgenic AR112Q muscle. Skeletal muscle from knockin AR113Q mice display decreased proteasomal activity (71). This finding, together with similar increases in PGD between the transgenic AR112Q and knockin AR113Q muscle, suggests that decreased proteasomal activity may be conserved between the 2 models of SBMA and may contribute to the imbalance observed in the proteomics. In addition, aged skeletal muscle also displays a similar proteomic imbalance and decreased proteasomal activity (72, 73).

We next performed gene ontology (GO) enrichment analysis of the differentially expressed proteins to gain insights into the biological processes, molecular functions, cellular components, and pathways that were altered in transgenic AR112Q quadriceps muscle (Figure 6E). The terms for each category were ranked based on adjusted *P* < 0.05 with the top 15 terms displayed (full GO analysis in Supplemental Table 3). In line with the observed changes in metabolites, upregulated proteins were enriched for terms associated with NADP metabolism, the pentose phosphate pathway, oxidoreductase activity, and mitochondria. In addition, we found that all categories were enriched for terms associated with the endoplasmic reticulum and Golgi apparatus, including vesicular transport between the 2 organelles. In line with data identifying altered expression of genes regulating muscle structure and contraction across several SBMA mouse models (74), our GO analysis was enriched for terms related to cytoskeletal and contractile elements of the muscle fiber, including actin and elements of the sarcomere. GO analysis of downregulated proteins showed enrichment for terms associated with spermine biosynthetic process and glucose catabolic process to lactate via pyruvate (Supplemental Table 3).

Together, these data suggest alterations in several metabolic pathways that rely on NAD^+^ metabolism and are important for energy production and cellular health; these include the pentose phosphate pathway, which is essential in the reduction of NADP^+^ to NADPH to protect cells from oxidative stress, and glucose catabolism, which is important for the production of ATP through glycolysis. In addition, our proteomics analysis also reveals changes that suggest alterations related to the ER, the Golgi apparatus, and transport between the two.

### NAD^+^-dependent pathways are enriched in metabolite/protein joint pathway analysis.

**Figure 7:**
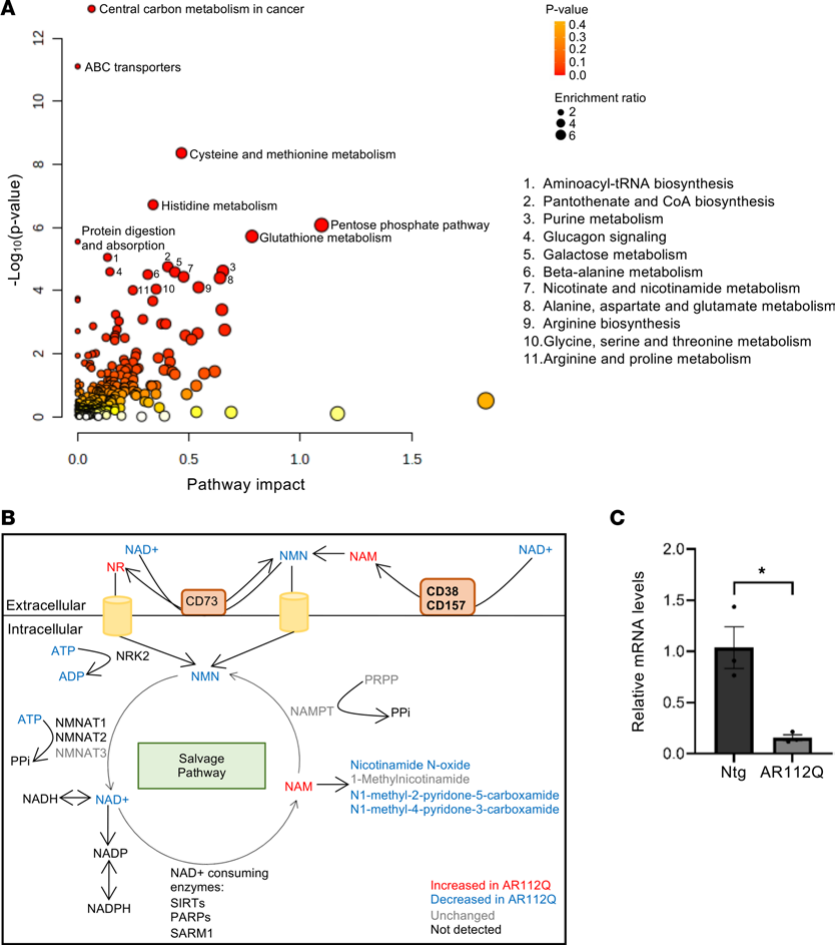
The NAD^+^ salvage pathway is altered in AR112Q quadriceps muscle. (**A**) Joint pathway analysis determined from combined metabolomic and proteomic alterations identifies significantly altered metabolic pathways that include nicotinamide metabolism and several other pathways that rely on nicotinamide metabolism, including pentose phosphate pathway, glutathione metabolism, and amino acid metabolism. Dot sizes increase with the increasing pathway impact; color intensifies according to the *y* axis. (**B**) Pathway diagram of NAD^+^ salvage pathway. Metabolic intermediates and proteins depicted in red were found to be increased and blue were found to be decreased in our metabolomics and proteomic analysis of quadriceps muscles from AR112Q versus nontransgenic mice at 36 weeks of age. Black, proteins and metabolites undetected in quadriceps muscle. Gray, unchanged. NR, nicotinamide riboside; NMN, nicotinamide mononucleotide; NAM, nicotinamide; NRK2, nicotinamide riboside kinase 2; NAMPT, nicotinamide phosphoribosyl transferase; NMNAT1-3, nicotinamide nucleotide adenylyltransferases; PRPP, phosphoribosyl pyrophosphate; SIRT, sirtuins; PARPs, poly (ADP-ribose) polymerases; SARM1, sterile alpha and TIR motif containing 1. (**C**) Real-time qPCR analysis of quadriceps muscle for *Nmrk2*, the enzyme responsible for conversion of NR to NMN, with transcript levels normalized to *Cpsf2*. Statistical significance was determined by Student’s *t* test. Data represent mean ± SEM. *P ≤ 0.05, *n* = 3 per group.

We next integrated the data from the metabolomics and proteomics data sets using a joint pathway analysis to identify critical pathways that may be impacted by both metabolomic and proteomic changes. Significant pathways with the highest impact included nicotinamide metabolism and several other pathways that rely on products of nicotinamide metabolism, including pentose phosphate pathway, pyruvate metabolism, glutathione metabolism, and amino acid metabolism (Figure 7A and Supplemental Table 4). While our proteomics data do not include posttranslational modifications, these data, together with the decrease in NAD, suggest dysfunction in pathways involving nicotinamide metabolism, including the NAD^+^ salvage pathway, where all involved metabolites are altered (Figure 7B and Supplemental Figure 4).

In muscle, when NR enters the cell, it is phosphorylated by the enzyme nicotinamide riboside kinase 2 (NRK2) (48), resulting in its conversion to nicotinamide mononucleotide (NMN), which is then able to enter the NAD^+^ salvage pathway to produce NAD^+^. NRK2 was not detected in the proteomics data set, leading us to specifically investigate its levels in quad of AR112Q mice. Given the absence of a commercially available antibody against NRK2, we utilized quantitative reverse transcription PCR (qRT-PCR) to evaluate *Nmrk2* mRNA levels. Expression of *Nmrk2* was significantly decreased in transgenic AR112Q muscle compared with nontransgenic muscle (Figure 7C). Together, these data suggest a model in which NAD^+^ levels are significantly decreased, potentially due to altered salvage pathway activity. In addition, NR entry into the salvage pathway is severely impacted by the decreased levels of *Nmrk2*, resulting in the lack of effect of NR dietary supplementation.

## Discussion

In order to effectively treat postsymptomatic SBMA, potential therapies must attenuate the toxicity of the mutant AR while ameliorating wide-ranging downstream metabolic symptoms. We have previously shown that SIRT1 overexpression reduces the aggregation and toxicity of polyQ-expanded AR by deacetylating the AR at 3 lysine residues in the hinge region (25). Moreover, activation of SIRT1 is protective against metabolic disorders known to affect patients with SBMA, such as nonalcoholic fatty liver disease (12, 75, 76), impaired glucose homeostasis (12, 13, 15, 36, 70), and mitochondrial dysfunction (35, 52, 65). Thus, there is ample evidence to suggest that increasing activation of SIRT1 could be an effective therapy for SBMA through both AR-specific and nonspecific mechanisms. To test this hypothesis, we treated postsymptomatic SBMA mice with the NAD^+^ precursor NR, finding that such dietary supplementation was unable to alter disease progression. Further investigation of energy metabolism in SBMA mice showed that ATP and NAD^+^ content were reduced in the muscle but not in the spinal cord, revealing tissue-specific metabolic disruption in this mouse model. Moreover, NAD^+^ and ATP levels in the muscle were not restored by treatment with NR, potentially due to decreased levels of the NR phosphorylating kinase NRK2 impacting NR use by the salvage cycle. In transgenic AR112Q muscle, NR treatment did result in increases in NAD^+^, NMN, and NAM clearance products compared with transgenic animals on the control diet. However, NAD^+^ levels after NR supplementation were still markedly lower than those of nontransgenic animals, suggesting that some of the NR was being utilized but that it was insufficient to make an impact on the substantially decreased NAD^+^ levels.

The precise mechanism by which NAD^+^ is reduced in SBMA muscle is unclear, but it is known that decreasing NAD^+^ in skeletal muscle disrupts muscle function and energy metabolism in mice (60). To gain a better understanding, we generated comprehensive metabolomic and proteomic profiles, focusing on mice fed the control diet, in which we observed that metabolites involved in the NAD^+^ salvage pathway were altered in AR112Q quad, suggesting either reduced NAD^+^ recycling via the salvage pathway or increased NAD^+^ consumption. While there was no change in levels of the key salvage pathway rate-limiting enzyme NAMPT, this does not rule out changes in activity that may be due to alterations in NAMPT posttranslational modifications. Recent work in symptomatic transgenic AR100Q quad also showed decreased levels of NAD^+^ in addition to decreased levels of the NAD^+^ consuming enzymes SIRT3 and PARP1, suggesting increased NAD^+^ consumption is not responsible for the decrease in NAD^+^ in the AR100Q mouse (77). More research is needed to elucidate the mechanisms contributing to decreased NAD^+^ levels in SBMA muscle. AR112Q muscle also showed evidence of altered glucose metabolism, including accumulation of several downstream glycolytic intermediates, with nodes of NAD^+^ dependence, and decreased levels of several glycolytic enzymes (Supplemental Figure 6B). In addition, points of regulation of TCA enzymes by NAD^+^ exhibited increased metabolites preceding that step (isocitrate and malate), suggesting impairment of the TCA cycle (Supplemental Figure 6A). The pentose phosphate pathway, the main producer of NADPH utilized in combating ROS, also displayed an accumulation of an important metabolite, 6-phosphogluconate, an intermediate that is NADP^+^ dependent (Supplemental Figure 6C). NAD^+^ is needed to make NADP^+^, on which several steps within, and upstream of, the pentose phosphate pathway rely. Thus, a decrease in NAD^+^ content could lead to dysregulation of a variety of NAD^+^-dependent processes, including glycolysis, the TCA cycle, and antioxidant pathways, several of which appeared in our pathway enrichment analysis in the muscles of SBMA mice, impacting both energy production and muscle function.

In the present study, supplying mice with the NAD^+^ precursor NR did not ameliorate metabolic or behavioral phenotypes. Although several studies have shown that dietary supplementation with 400 mg/kg/d NR effectively boosts NAD^+^ in the muscle of mice (39, 50, 53), the lack of effects observed in our results is most likely due to decreased NRK2 in transgenic quad, thereby limiting the phosphorylation of NR and preventing its entry into the NAD^+^ salvage pathway. Other studies have reported limitations in the use of orally delivered NR to boost NAD^+^ in skeletal muscle, due to NR conversion to NAM in the liver, prior to entering circulation, while intravenously delivered NR was directly converted to NAD^+^ in the muscle (60, 78). Metabolism of orally delivered NR into NAM prior to entering circulation could mitigate its ability to increase NAD^+^ if the activity of NAMPT is impaired in target tissues. Moreover, conditional knockout of NAMPT in the projection neurons of adult mice and muscle-specific NAMPT deletion in mice recapitulates a neuromuscular disease phenotype, causing motor neuron degeneration, motor function deficits, mitochondrial dysfunction, muscle fiber type switching, and disruption at neuromuscular junctions (60, 79, 80). In SBMA muscle, NAM levels were increased while NMN levels were decreased. While NAMPT levels were not altered in our data set, this does not rule out possible changes in NAMPT activity, which could result in reduced conversion of NAM to NMN and a reduction in NAD^+^ levels. Therefore, the reason NR treatment failed to have an effect in this study may be a combination of several processes, including the inefficient phosphorylation of NR and an already compromised NAD^+^ salvage pathway unable to utilize NAM. Future studies will be necessary to determine the mechanism underlying the observed decrease in NAD^+^ levels and alterations in NAD^+^ salvage pathway metabolites in SBMA skeletal muscle.

It is likely that increasing NAD^+^ levels in SBMA with the ideal NAD^+^ precursor and/or delivery method could have a protective effect. This remains a tantalizing therapeutic prospect, as human trials have already determined that chronic NR supplementation is well tolerated in older adults (81) and effectively stimulates NAD^+^ metabolism in a dose-dependent manner (82). Supplementation with NMN could be a potential therapy as it is also well tolerated and effective in mice and can enter the NAD^+^ salvage pathway downstream of NAMPT via the recently described transporter, SLC12A8 (83–85). Moreover, identifying a compound that can increase NAD^+^ levels in muscle could be a potential therapeutic for SBMA, since selective targeting of skeletal muscle in SBMA mice ameliorated the hallmarks of disease (4, 86) and highlighted the importance of skeletal muscle in both the pathogenesis and treatment of SBMA.

This study establishes that treatment of postsymptomatic SBMA mice with the NAD^+^ precursor NR is unable to rescue behavioral or metabolic phenotypes. Furthermore, our investigation of energy metabolism in the spinal cord and muscle of SBMA mice revealed a striking difference in the downstream effects of polyQ-expanded AR expression in each of these tissues. The relative lack of observed metabolomics alterations in spinal cords of SBMA mice likely reveals true tissue-specific differences in this model. Several metabolomics analyses of spinal cords from mouse models of ALS have revealed substantive alterations in the disease state (87, 88), suggesting that the lack of observed metabolomics alterations between transgenic and nontransgenic animals in our study was unlikely to be caused by a dilution effect of analyzing whole spinal cord. Further experimentation will be necessary to determine which step(s) of NAD^+^ production are impaired in the muscle of SBMA mice, whether these processes remain intact in the CNS, and if, within the CNS, motor neurons are specifically vulnerable to disrupted energy homeostasis. Finally, we found that AR112Q mice exhibit reduced blood glucose levels, a newly characterized phenotype that may reflect the impaired glucose homeostasis observed in patients with SBMA. Together, these experiments highlight the tissue specificity of metabolic disruptions in SBMA and provide useful insights into the potential and limitations of dietary NAD^+^ precursor supplementation in the treatment of neuromuscular disease.

## Methods

### Sex as a biological variable.

Our study exclusively examined male mice because SBMA only affects males.

### Animals.

Transgenic mice expressing human AR112Q under the control of the PrP promoter were previously generated as described (57).

Animal cages were randomly assigned to a treatment group using a random number generator. Mice were fed normal chow (Purina Rodent Chow 5001) or normal chow supplemented with NR (ChromaDex) at a concentration of 2.4 g NR/kg chow, prepared by Research Diets. This concentration was based upon previously published data (and our unpublished observations) on the food intake of C57BL/6J mice (89) in order to treat mice with 400 mg/kg/d NR. NR treatment began at 10 weeks immediately following behavioral testing. Mouse chow was coded to prevent user knowledge of treatment group. Mice were given ad libitum access to food and water.

### Mouse genotyping.

DNA was extracted from mouse ear biopsies using REDExtract-N-Amp Tissue PCR kit (MilliporeSigma). Transgenic animals were identified by PCR of the human AR: forward primer from the PrP promoter region (5′-ACTGAACCATTTCAACCGAGC-3′) coupled with a reverse primer from the AR sequence (5′-AGGTGCTGCGCTCGCGGCCTCT-3′).

### Analysis of motor function.

The following cohorts of age-matched, male mice were evaluated for motor function: AR112Q mice fed control chow (*n* = 26), AR112Q mice fed NR-supplemented chow (*n* = 24), nontransgenic mice fed control chow (*n* = 24), and nontransgenic mice fed NR-supplemented chow (*n* = 26). Behavioral testing began at 10 weeks of age and was carried out every 4 weeks for 24 weeks. All behavioral testing was carried out by investigators blinded to animal genotypes and treatment group. Behavioral testing occurred during the light phase of a 12-hour light/12-hour dark cycle.

Analysis of motor coordination was performed using a continuously accelerating (4–40 rpm over 10 minutes) rotarod (UgoBasile). During the first week of testing (10 weeks of age), cohorts were trained with 4 trials per day for 3 consecutive days. Results from the third day were kept for analysis. In subsequent weeks of testing, mice performed 4 trials on a single day for a maximum duration of 400 seconds. Mice were removed from the rotarod if they rotated twice around the walking beam. Mice were rested for at least 30 minutes between trials.

Forepaw and all-paw grip strength were evaluated using a grip strength meter (Columbus Instruments). Six measurements were taken for each animal, and the highest and lowest data points were dropped. The remaining 4 values were averaged and used for statistical analysis.

### Histological analysis of muscle.

Quads were embedded on balsa wood in OCT and flash-frozen in liquid nitrogen–cooled isopentane prior to storage in liquid nitrogen. NADH-tetrazolium reductase–stained sections of the quads were examined histologically from nontransgenic and transgenic mice fed either a control diet or a diet supplemented with 400 mg/kg/d NR. Muscle fiber cross-sectional area and NADH staining intensity were measured using ImageJ (NIH). NADH staining intensity was determined by measuring the mean gray value of images collected with the same light intensity and exposure time.

### Immunostaining.

Immunostaining was carried out as described (58) following 20 minutesʼ fixation in 4% paraformaldehyde. Spinal cord tissue was embedded in OCT and cryosectioned (7 μm) prior to fixation.

Antibodies used include AR (H280, Santa Cruz Biotechnology) and neurofilament-heavy chain (SMI32; 801701 Sternberger Monoclonals). Immunostained spinal cord sections were visualized using a DMR Fluorescence microscope (Leica Microsystems) and imaged using ProgRes software.

### ATP and NAD^+^ measurement.

Flash-frozen spinal cord and quad were pulverized using mortar and pestle on dry ice prior to analyses. NAD^+^ was measured with the EnzyChrom NAD/NADH Assay Kit (E2ND-100, BioAssay Systems) according to manufacturer’s instructions. ATP was measured with the ATP Determination Kit (A22066, Thermo Fisher Scientific) from neutralized acid extracts prepared with the EnzyChrom NAD/NADH Assay Kit.

### H_2_O consumption.

Average H_2_O intake per mouse was determined by weighing the water bottle of each cage on consecutive days and dividing by the number of mice per cage (assuming 1 g/mL for the density of water). The average H_2_O intake of nontransgenic mice is in agreement with previously published data on the water intake of C57BL/6J mice (89).

### Blood glucose measurement.

Blood glucose measurements were taken from the tail vein of mice using the AlphaTRAK 2 glucose meter (Zoetis) immediately following euthanasia by CO_2_ at 36 weeks.

### Untargeted global metabolomic analysis.

Quad of 9-month-old transgenic AR112Q and nontransgenic mice fed either a control or NR-supplemented diet (*n* = 7 per group) were used for metabolomics analysis. Upon euthanasia, quads were rapidly dissected and snap-frozen in liquid nitrogen. Metabolomic profiling was performed by Metabolon Inc. according to previously published methods (90, 91). In brief, proteins were removed from samples and prepared according to a proprietary series of organic and aqueous extractions. The extracted samples were then analyzed on the gas chromatography/MS and LC-MS/MS platforms. For LC-MS/MS, samples were split and analyzed in both the positive and negative ionization mode. Biochemicals were compared to the reference library, and missing values were imputed with the observed minimum value. Significantly changed metabolites were based on *P* ≤ 0.05, false discovery rate of *q* < 0.1, and a metabolite ratio of >1.0 for increased and <1.0 for decreased metabolites.

### MSEA and joint pathway analysis.

The web-based metabolomic data processing tool, MetaboAnalyst 5.0 (http://metaoanalyst.ca) (92, 93), was used for MSEA and joint pathway analysis. MSEA was used to identify overrepresented metabolic pathways in transgenic PrP-AR112Q quad compared with nontransgenic mice fed the control diet. For MSEA, significantly dysregulated metabolites were uploaded and mapped according to the Human Metabolome Database. The hypergeometric test was used to evaluate if a metabolite set was overrepresented. After correcting for multiple testing by applying a false discovery rate of 10%, 1-tailed *P* values were displayed. The enrichment ratio was calculated by observed hits/expected hits.

The joint pathway analysis was used to identify metabolic pathways of interest by analyzing both significantly changed metabolites and proteins based on the metabolic pathway database Kyoto Encyclopedia of Genes and Genomes containing both metabolites and metabolic genes. Parameters for the algorithm included the hypergeometric test for the enrichment analysis, degree centrality for the topology measure, and combine queries as the integration method. The overview of pathway analysis is created using the *P* values from the pathway enrichment analysis and the pathway impact values from the pathway topology analysis.

### Proteomic analysis.

For sample preparation, animals (3 per genotype) were euthanized at 9 months of age. Upon euthanasia, animals were quickly dissected, and quads were excised and snap-frozen in liquid nitrogen. Snap-frozen quads were then pulverized on dry ice, 10 times the tissue weight of lysis buffer (50 mM Tris at pH 7.5, 150 mM NaCl, 1 mM EDTA, 150 μM PMSF, 1% SDS, protease and phosphatase inhibitors) was added, and sample was homogenized with a LABGEN 850 Homogenizer 110 V (Cole-Parmer) at 13,000*g* 3 times for 20 seconds. Lysate was then sonicated and centrifuged at 13,000*g* for 15 minutes. Protein concentration was determined using DC assay (Bio-Rad). A total of 25 μg of total protein lysate was electrophoresed 1 cm into a 10% SDS-PAGE (Bio-Rad) and stained with Collodial Coomassie blue (Thermo Fisher Scientific).

The entire stained gel lanes were excised, reduced with TCEP, alkylated with iodoacetamide, and digested with trypsin (Promega). LC-MS/MS was performed using a Q Exactive HF mass spectrometer (Thermo Fisher Scientific) coupled with an UltiMate 3000 nano UPLC system (Thermo Fisher Scientific). Samples were injected onto a PepMap100 trap column (0.3 × 5 mm, packed with 5 μm C18 resin; Thermo Fisher Scientific), and peptides were separated by reversed-phase HPLC on a BEH C18 nanocapillary analytical column (75 μm inner diameter × 25 cm, 1.7 μm particle size; Waters) using a 4-hour gradient formed by solvent A (0.1% formic acid in water) and solvent B (0.1% formic acid in acetonitrile). Eluted peptides were analyzed by the mass spectrometer set to repetitively scan *m/z* from 400 to 1,800 in positive-ion mode. The full MS scan was collected at 60,000 resolution followed by data-dependent MS/MS scans at 15,000 resolution on the 20 most abundant ions exceeding a minimum threshold of 20,000. Peptide match was set as preferred, exclude isotopes option was enabled, and charge-state screening were used to reject unassigned, single, and more than 7 charged ions.

Peptide and proteins were identified using MaxQuant 1.6.17.0 (94). MS/MS spectra were searched against a Swiss-Prot mouse protein database (July 2021) and a common contaminants database using full tryptic specificity with up to 2 missed cleavages, static carbamidomethylation of Cys, and variable Met oxidation, protein N-terminal acetylation, and Asn deamidation. “Match between runs” feature was used to help transfer identifications across experiments to minimize missing values. Consensus identification lists were generated with FDR set at 1% for protein and peptide identifications. Statistical analyses were performed using Perseus 1.6.15 (95). Missing values were imputed with a minimum value, and Student’s 2-tailed *t* test *P* values were adjusted to account for multiple testing using permutation-based FDR function in Perseus.

### GO analysis.

GO enrichment analysis was performed using the open source online PANTHER classification system (http://pantherdb.org/; version 17.0) to determine the major biological processes, molecular functions, cellular components, and pathways that were overrepresented by the differentially abundant proteins. Test type was Fisher’s exact with FDR correction.

### Western blotting.

Western blotting was performed on the same quad lysate used for the mass spectrometry analysis (*n* = 3). Equal amounts of protein from each sample were separated using the TGX stain-free SDS-PAGE system from Bio-Rad. Proteins were then transferred onto a PVDF membrane, blocked in 0.1% TBS-Tween 20 with 5% milk, and probed with the following primary antibodies: FASN (1:1,000, Cell Signaling Technology 3180), PFKM (1:1,000, Abcam ab154804), PGD (1:1,000, Abcam ab129199), and PGM1 (1:1,000, Abcam ab192876). Proteins were visualized using a chemiluminescence system (Bio-Rad) and normalized to total protein.

### qRT-PCR.

Frozen quad was first pulverized on dry ice and then homogenized in TRIzol (Thermo Fisher Scientific), and RNA was extracted by isopropanol precipitation from the aqueous phase. cDNA was synthesized with 1 μg of RNA using the SuperScript III first-strand synthesis system for RT-PCR kit (Invitrogen) per the manufacturer’s instructions. qRT-PCR was carried out using FastStart TaqMan Probe Master Mix (Thermo Fisher Scientific) on software supplied with a Quant Studio 3 system using FAM-labeled probes specific for *Nmrk2* and *Cpsf*2 (Thermo Fisher Scientific) (*Nmrk2*, Mm01172899_g1; *Cpsf2*, Mm00489754_m1). Relative expression levels were calculated by normalizing to the expression of *Cpsf2*.

### Statistics.

For pairwise comparisons, statistical significance was determined using Student’s 2-tailed *t* test. For multiple comparisons, statistical significance was determined using 2-way ANOVA with post hoc Tukey test or using mixed effects analysis with post hoc Tukey test for multiple comparisons. The significance level (α) was set at 0.05 for all experiments unless otherwise noted in figure legends. Data were compiled and analyzed using Microsoft Excel or Prism 9.5 (GraphPad).

### Study approval.

All animal procedures were performed following the guidelines of the Office of Laboratory Animal Welfare and with the approval of the Thomas Jefferson University Institutional Animal Care and Use Committee.

### Data availability.

Values for all data points in graphs are reported in the Supporting Data Values file.

## Author contributions

The experiments were conceived and analyzed by FJA, DD, HYT, and DEM. FJA, DD, EM, HYT and YL conducted assays and analyzed data. FJA and DD prepared figures. DD, FJA, HYT, EM, and DEM wrote the manuscript. The co–first author order was determined by the relative contributions of DD and FJA, with both involved in leading distinct aspects of the project.

## Supplementary Material

Supplemental data

Unedited blot and gel images

Supplemental table 1

Supplemental table 2

Supplemental table 3

Supplemental table 4

Supporting data values

## Version 1 03/07/2024

In-Press Preview

## Version 2 04/08/2024

Electronic publication
