# Supplementary material for: Differentially disrupted spinal cord and muscle energy metabolism in spinal and bulbar muscular atrophy
Source: JCI Insight. 2024 Mar 7;9(7):e178048. doi: 10.1172/jci.insight.178048 (PMC11128210; doi:10.1172/jci.insight.178048)
Supplement: Supplemental data [file jciinsight-9-178048-s090.pdf]

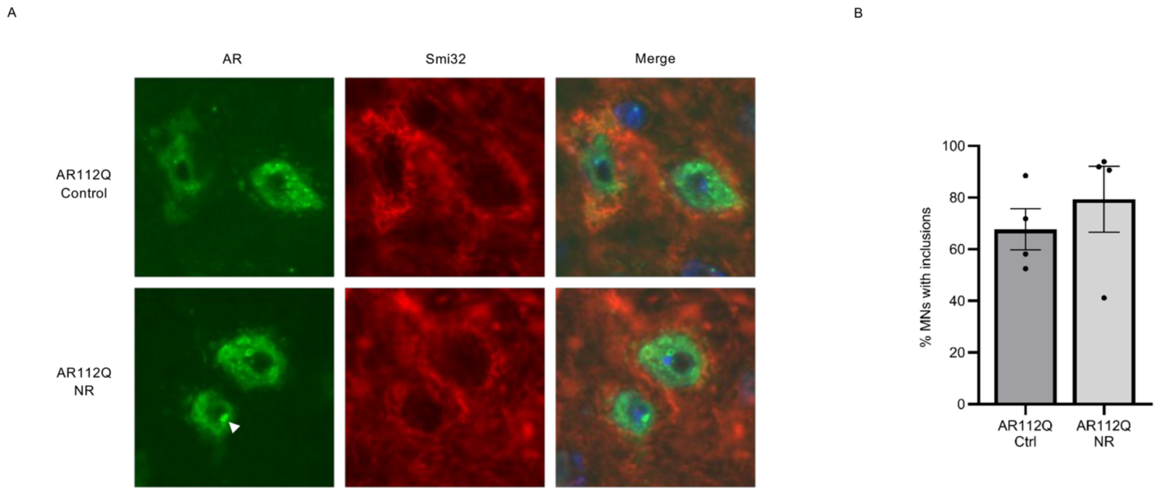

Supplemental Figure 1. NR treatment does not affect AR aggregation in SBMA mice.
(**A**) Immunofluorescence staining of motor neurons from spinal cord sections of control and NR-treated AR112Q mice at 36 weeks, using an anti-AR antibody to detect nuclear inclusions (white arrow) and SMI32 (uNF-H) to mark the motor neuron soma. Note that merged images include Hoechst staining to mark nuclei. Images taken at 40x magnification. (**B**) Percentage of motor neurons from control (n = 4) or NR-treated (n = 4) AR112Q mice with AR nuclear inclusions at 36 weeks. 40-140 motor neurons were counted per mouse. Statistical significance was determined by Student's t-test. Graphs show mean ± SEM.

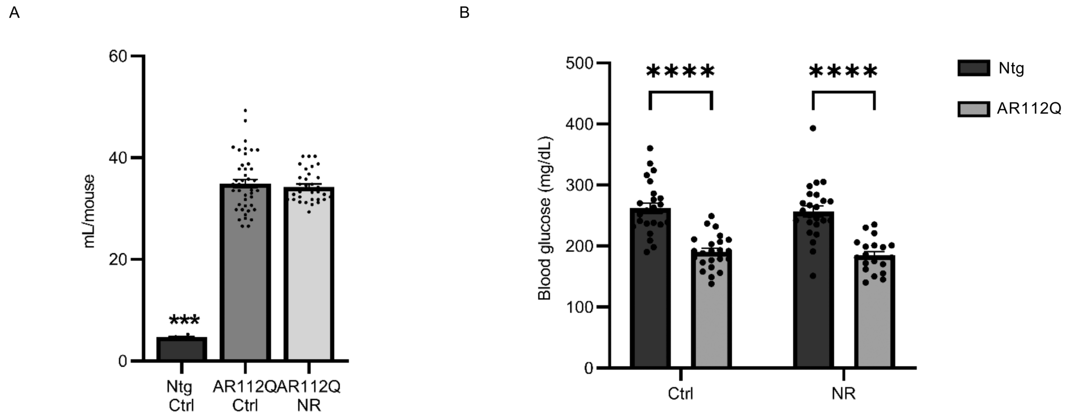

Supplemental Figure 2. PrP-AR112Q mice exhibit polydipsia and hypoglycemia.
(**A**) Average H_2O intake of non-transgenic and control or NR-treated AR112Q mice at 27 weeks. Measurements were taken on three consecutive days. Statistical significance was determined by one-way ANOVA with post hoc Tukey test. ***p<0.001. (**B**) Tail-vein blood glucose measurements of control or NR-treated non-transgenic and AR112Q mice at 36 weeks. Statistical significance was determined by one-way ANOVA with post hoc Tukey test. Data depict mean ± SEM ****p<0.0001.

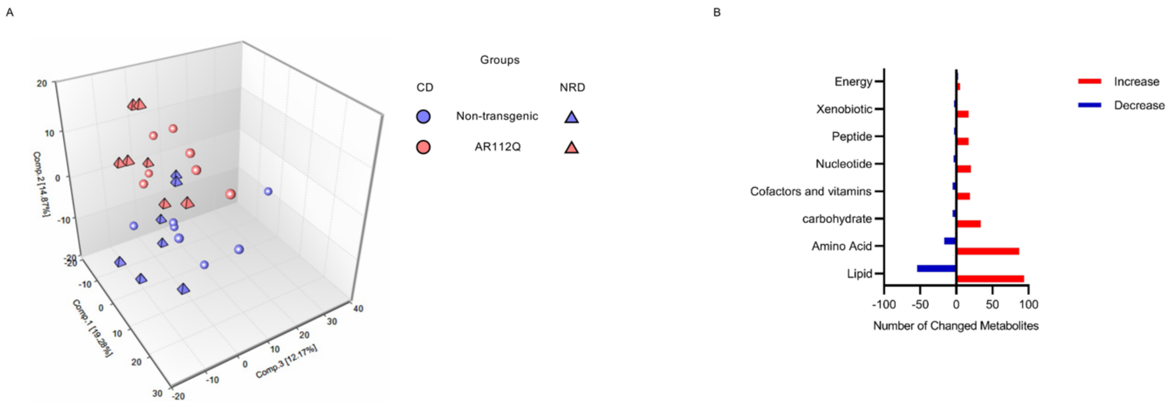

Supplemental Figure 3. Changed metabolites in AR112Q spinal cord and quadriceps muscle.

(**A**) Principle component analysis (PCA) of metabolomics datasets of spinal cord from AR112Q (pink symbols) and non-transgenic (blue symbols) mice, treated with NR diet (NRD) or control diet (CD). Plots of non-transgenic and AR112Q mice are not clearly distinguished from one another. n=7 per condition/genotype. (**B**) Metabolite class of significantly changed metabolites in the AR112Q quadriceps muscle treated with CD. n=7 mice per genotype.

	Tg/NTg		NRD/CD	
	CD	NRD	NTg	Tg
nicotinamide (NAM)	1.12	1.07	1.08	1.04
nicotinamide ribonucleotide (NMN)	0.29	0.85	1.03	3.00
nicotinamide riboside (NR)	1.74	1.95	0.99	1.11
nicotinamide adenine dinucleotide (NAD+)	0.01	0.18	0.86	15.76
nicotinamide N-oxide	0.78	1.49	6.10	11.56
1-methylnicotinamide	1.08	2.53	3.29	7.73
trigonelline (N'-methylnicotinate)	1.35	1.75	0.83	1.07
N1-Methyl-2-pyridone-5-carboxamide	0.78	0.93	4.19	4.97
N1-Methyl-4-pyridone-3-carboxamide	0.51	0.83	3.58	5.83
adenosine 5'-diphosphoribose (ADP-ribose)	0.77	3.33	0.66	2.86

Supplemental Figure 4. Changed NAD^+ salvage pathway metabolites in AR112Q quadriceps muscle.

Chart depicting the fold changes of significantly increased (red) or decreased (green) metabolites related to nicotinamide metabolism in quadriceps muscle of mice fed both the control (CD) and NR (NRD) diets. n=7 mice per genotype.

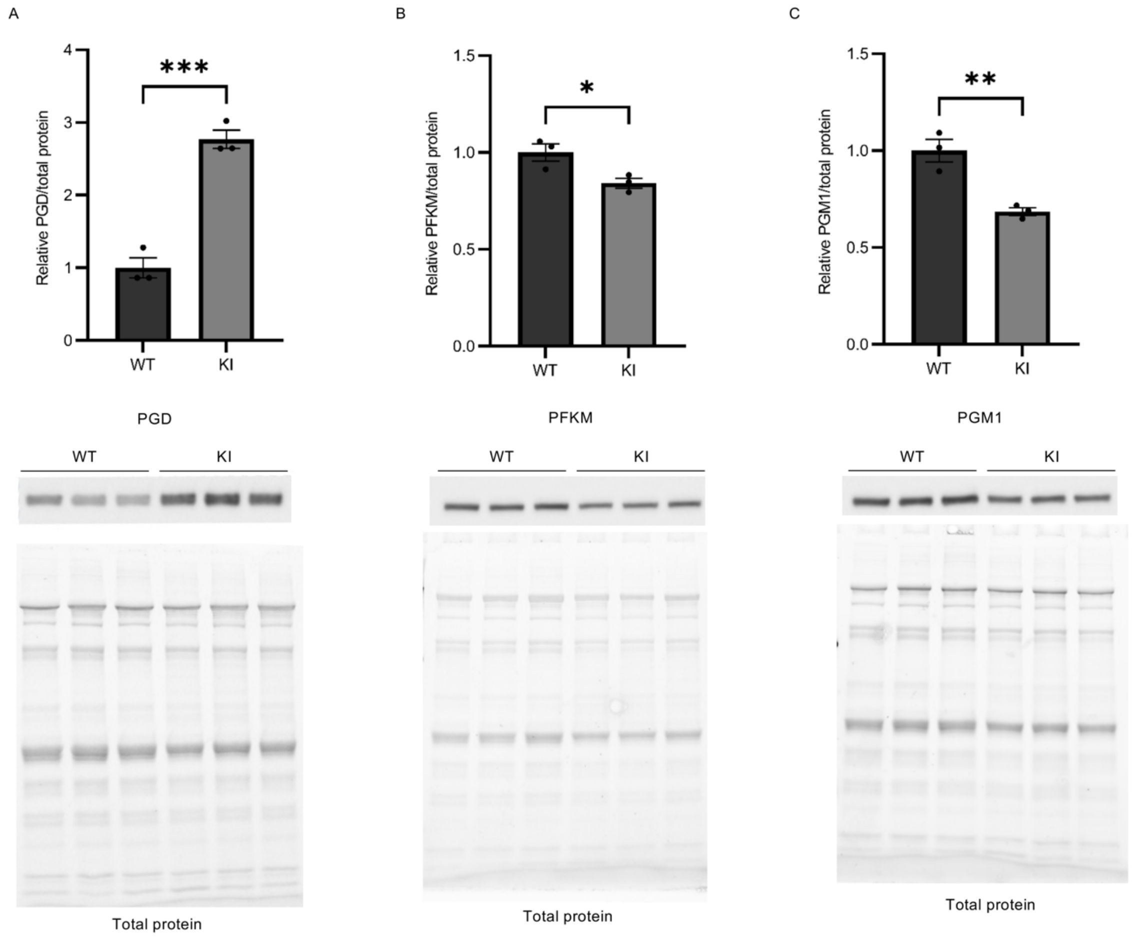

Supplemental Figure 5. Knock-in AR113Q quadriceps muscle shows similar proteomic changes to those observed in transgenic AR112Q mice (Fig. 6).
(A-C) Quantification and associated Western blots for selected proteins in knock-in AR113Q quadriceps muscle. Phosphogluconate dehydrogenase (PGD), phosphofructokinase (PFKM) and, phosphoglucomutase 1 (PGM1). Statistical significance was determined by Student's t-test. Graphs show mean ± SEM, n=3 per genotype. *** p≤0.001, **p≤0.01, *p≤0.05.

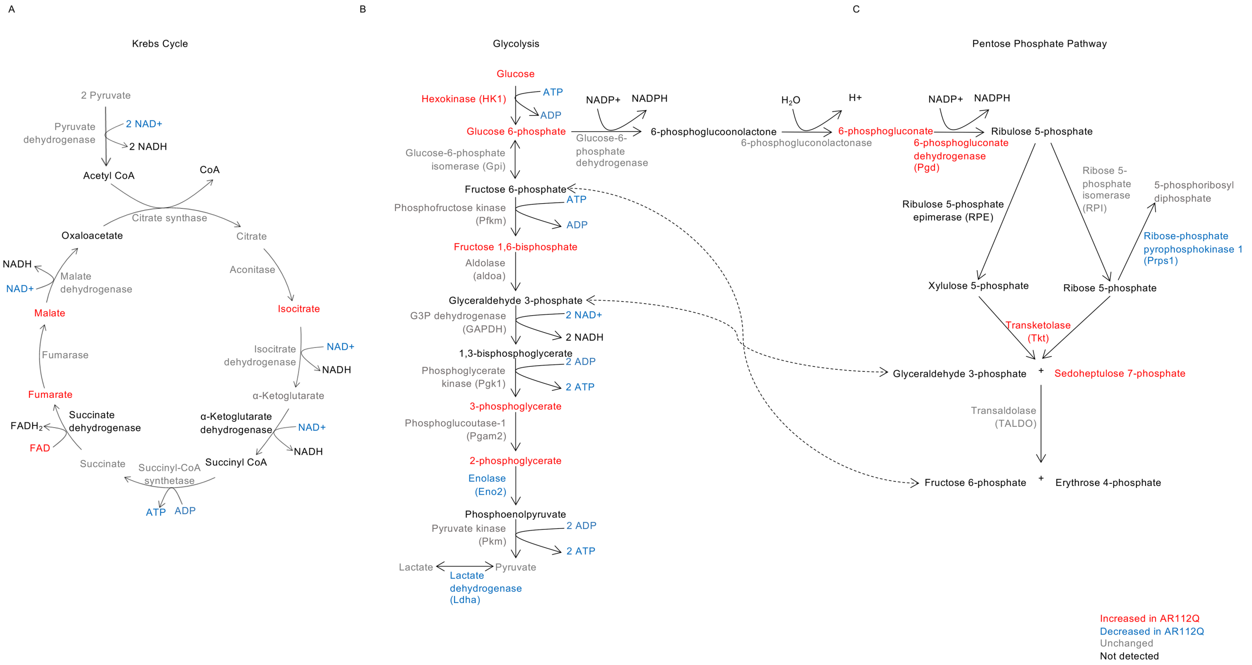

Supplemental Figure 6. Energy pathways impacted in AR112Q quadriceps muscle. Changed metabolites and proteins associated with the Krebs cycle (A), glycolysis (B) and the pentose phosphate pathway (C).
