# Supplementary material for: Differentially disrupted spinal cord and muscle energy metabolism in spinal and bulbar muscular atrophy
Source: JCI Insight. 2024 Mar 7;9(7):e178048. doi: 10.1172/jci.insight.178048 (PMC11128210; doi:10.1172/jci.insight.178048)
Supplement: Unedited blot and gel images [file jciinsight-9-178048-s091.pdf]

Full unedited gel for Figure 6A

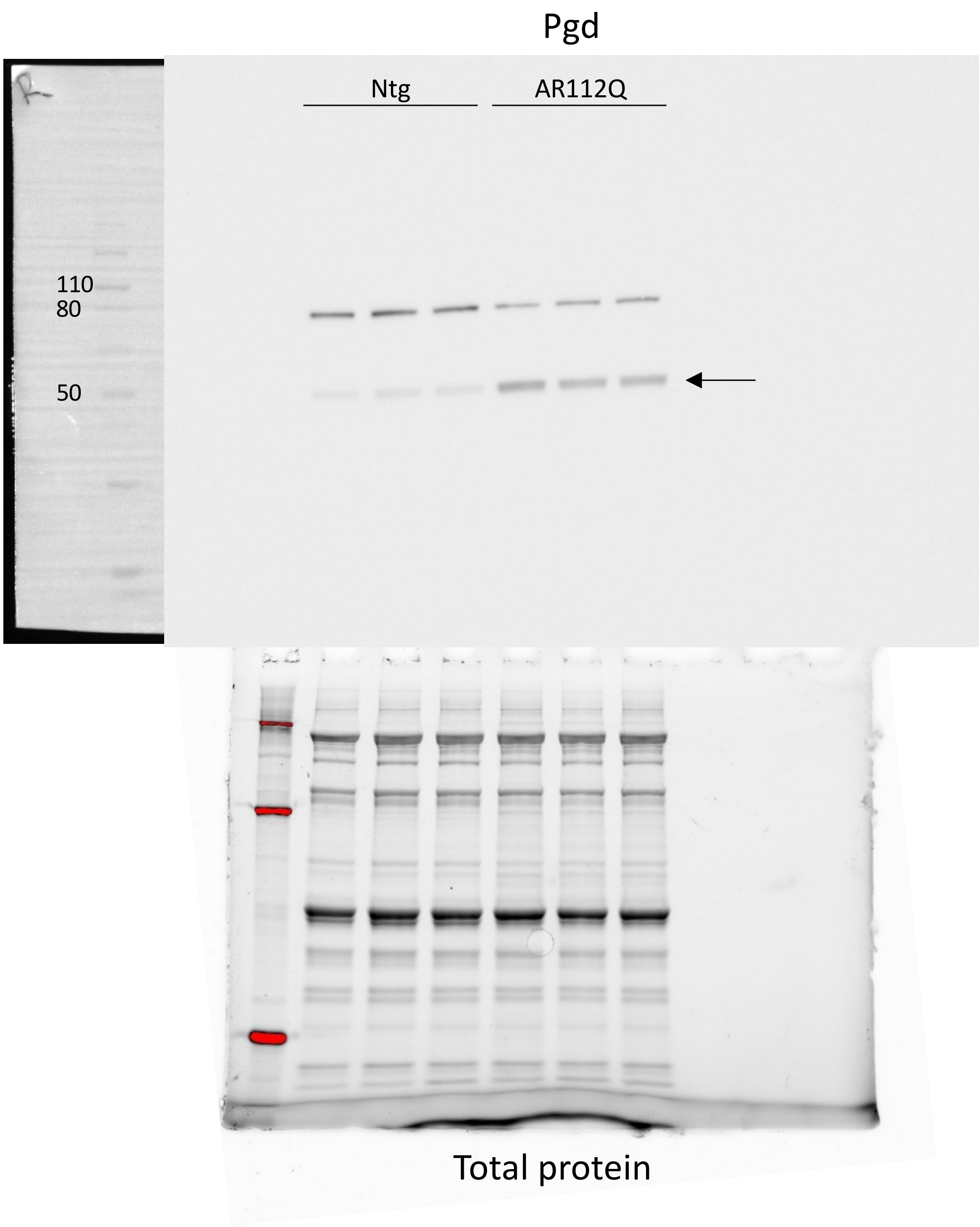

Full unedited gel for Figure 6B

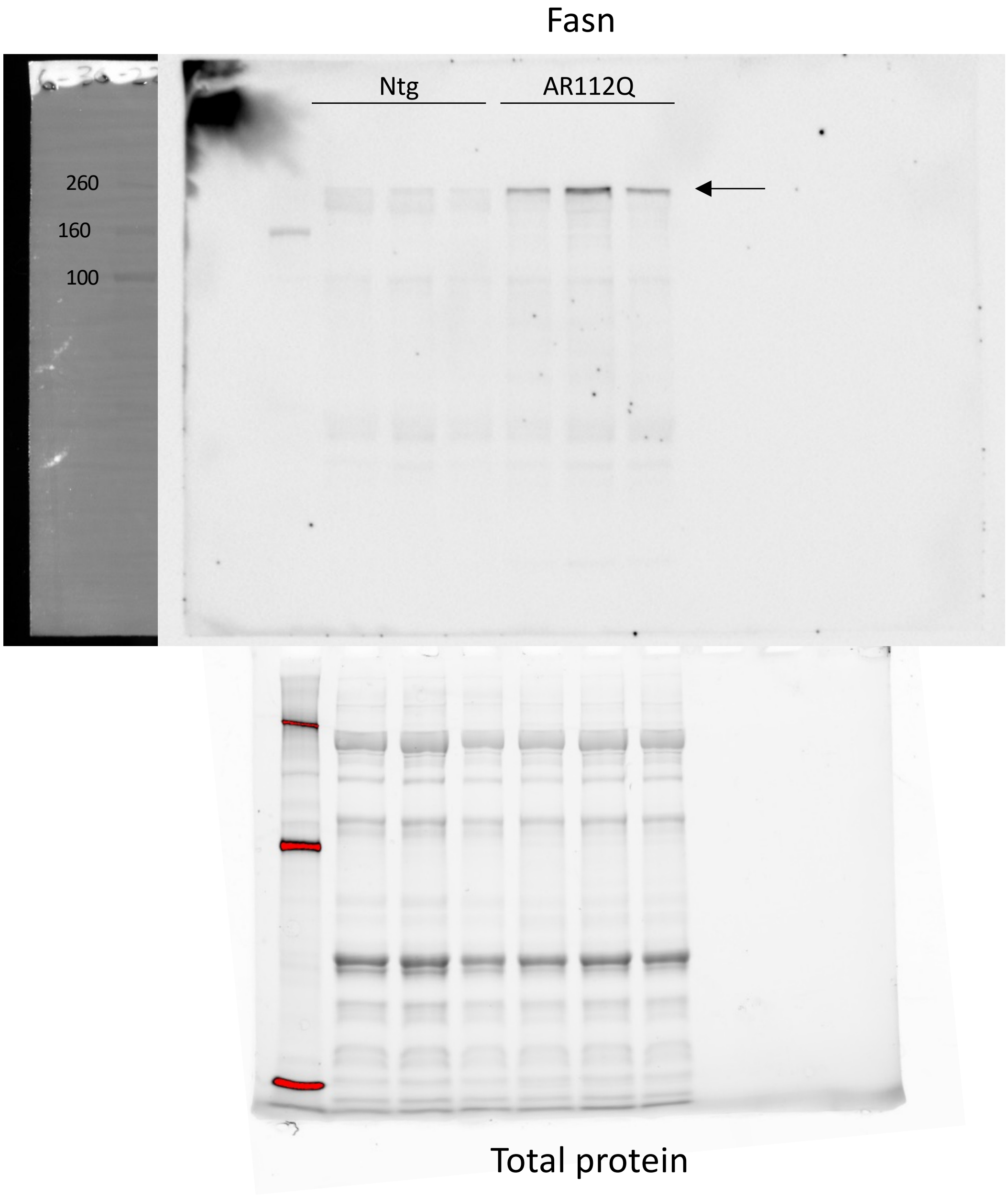

Full unedited gel for Figure 6C

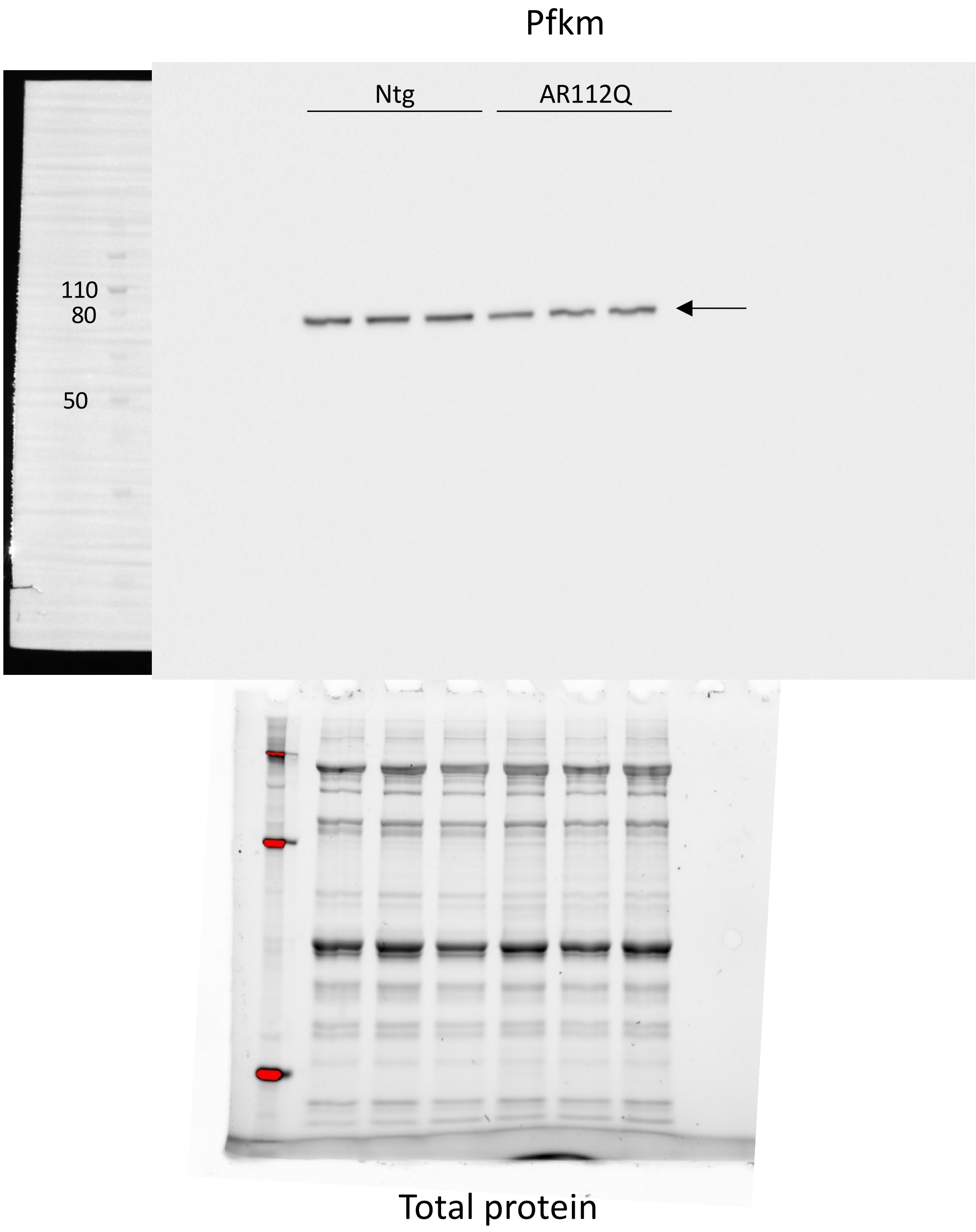

Full unedited gel for Figure 6D

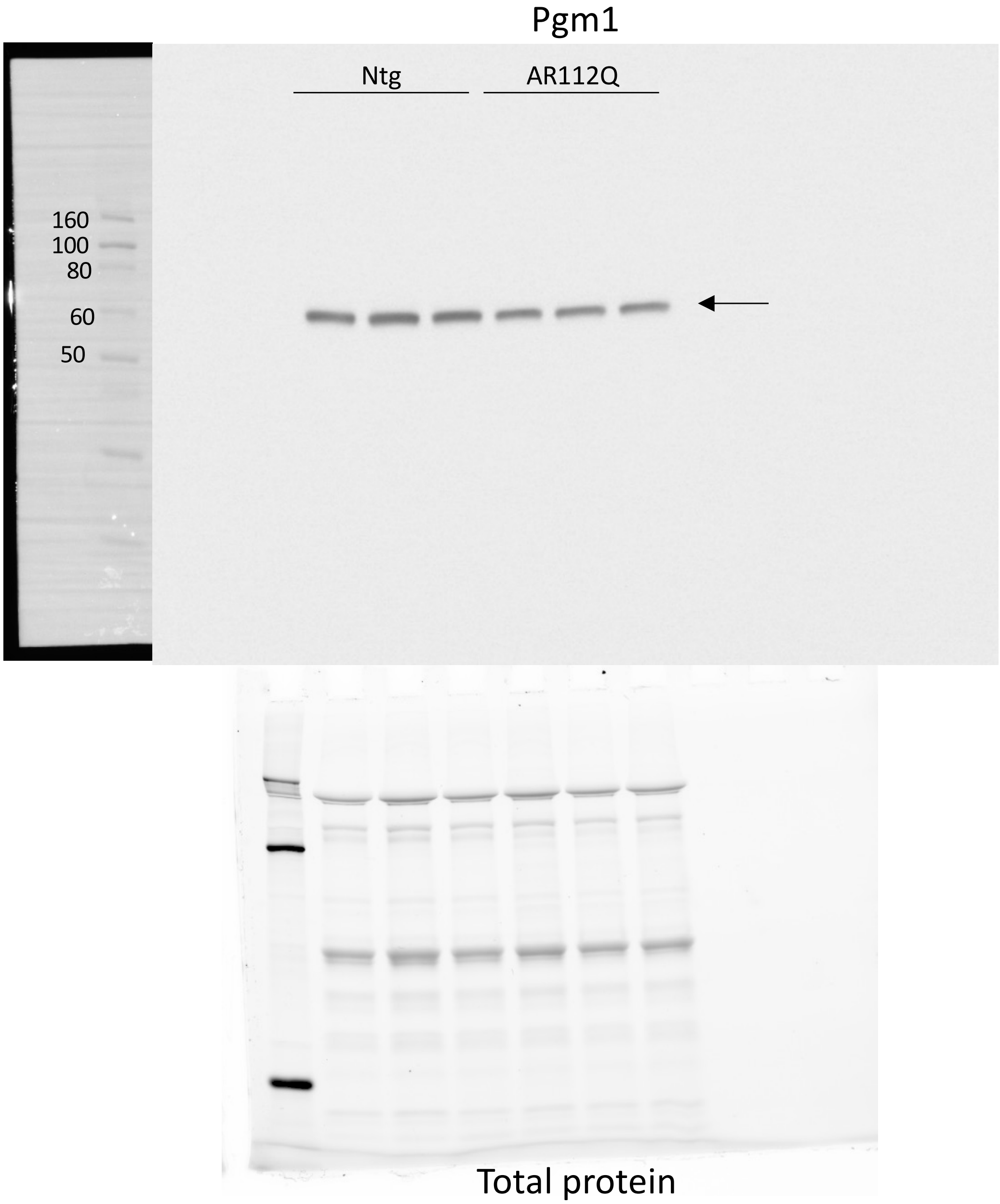

Full unedited gel for Supplemental Fig. 5A

Pgd

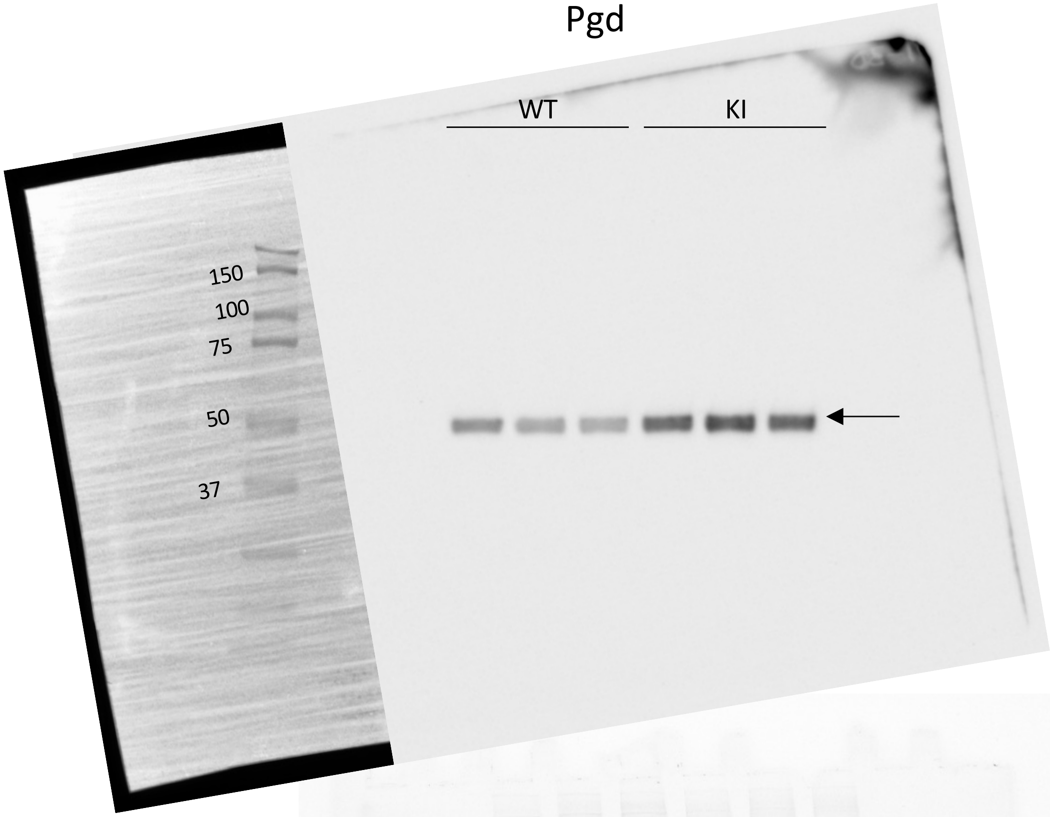

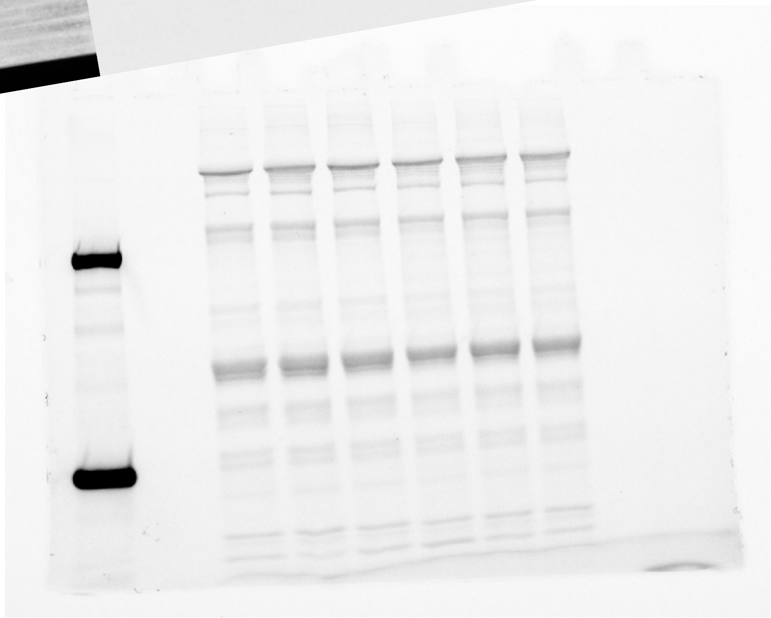

Total protein

Full unedited gel for Supplemental Fig. 5B

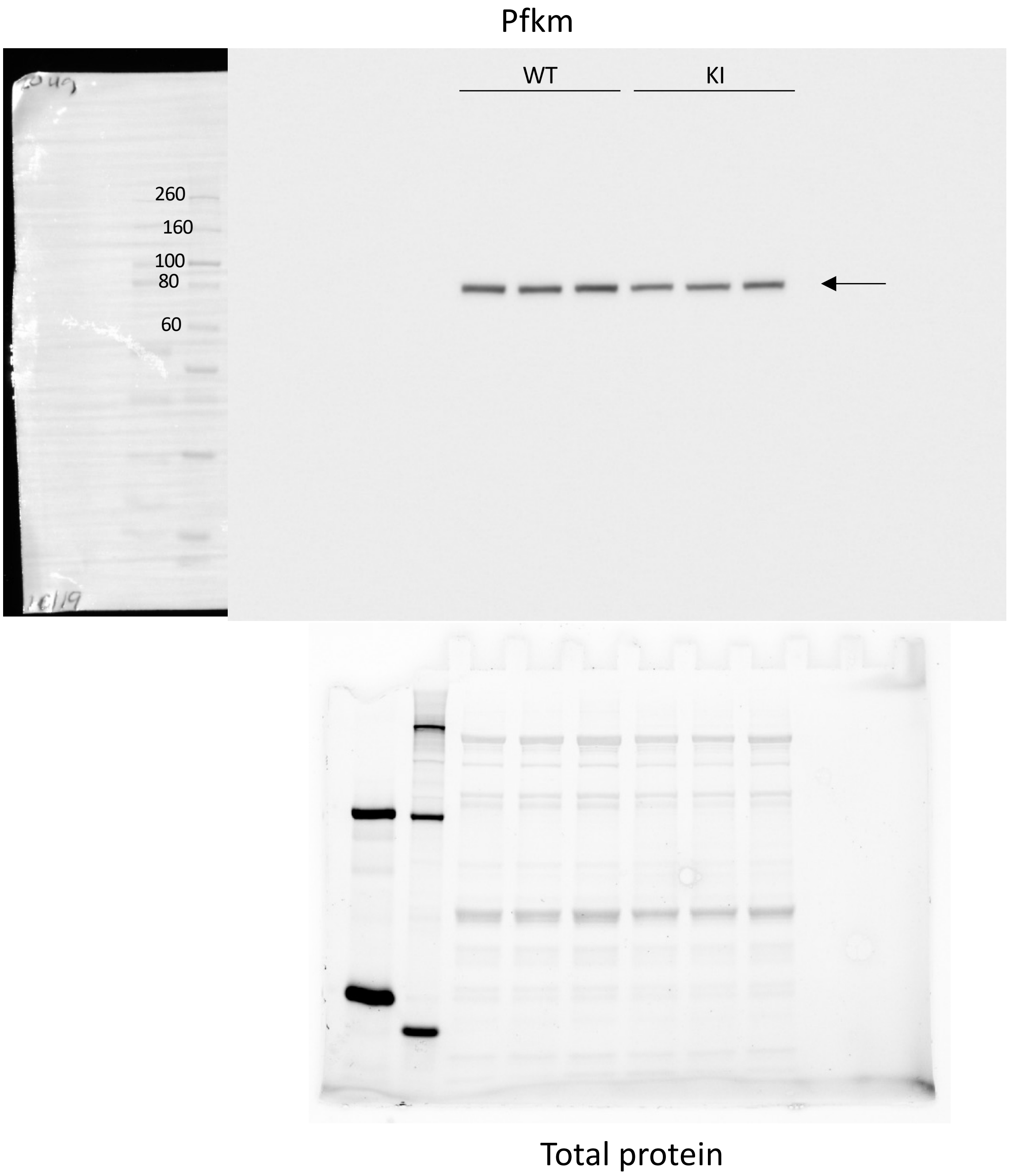

Full unedited gel for Supplemental Fig. 5C

Pgm1

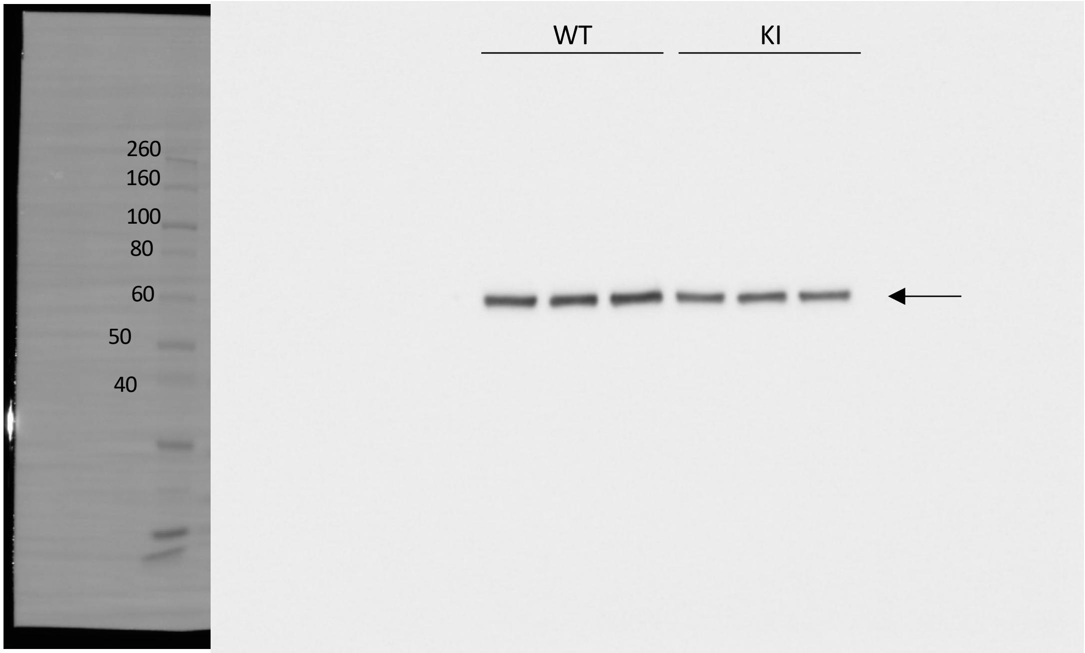

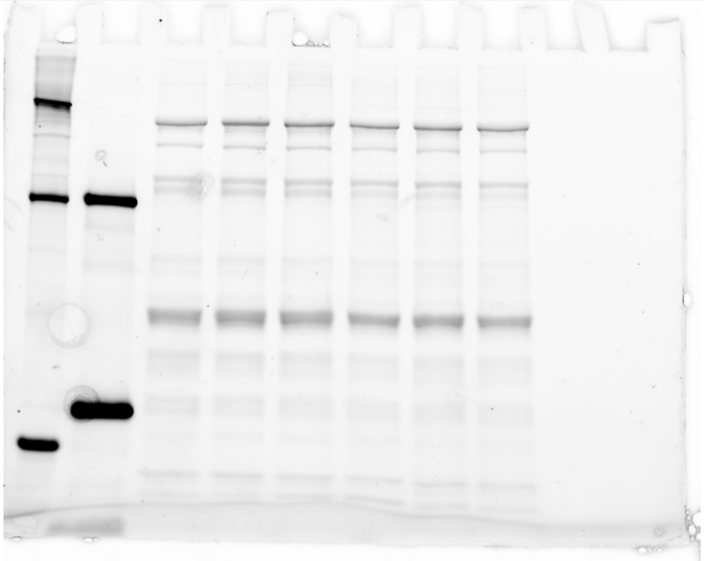

Total protein
